# Increased precipitation over land due to climate feedback of large-scale bioenergy cultivation

**DOI:** 10.1038/s41467-023-39803-9

**Published:** 2023-07-11

**Authors:** Zhao Li, Philippe Ciais, Jonathon S. Wright, Yong Wang, Shu Liu, Jingmeng Wang, Laurent Z. X. Li, Hui Lu, Xiaomeng Huang, Lei Zhu, Daniel S. Goll, Wei Li

**Affiliations:** 1 Department of Earth System Science, Ministry of Education Key Laboratory for Earth System Modeling, Institute for Global Change Studies, Tsinghua University, 100084 Beijing, China; 2 Laboratoire des Sciences du Climat et de l’Environnement, LSCE/IPSL, CEA-CNRS-UVSQ, Université Paris-Saclay, 91191 Gif-sur-Yvette, France; 3 Laboratoire de Météorologie Dynamique, Centre National de la Recherche Scientifique, Sorbonne Université, Ecole Normale Supérieure, Ecole Polytechnique, 75252 Paris, France; 4 Université Paris Saclay, CEA-CNRS-UVSQ, LSCE/IPSL, Gif sur Yvette, France; 5 Ministry of Education Ecological Field Station for East Asian Migratory Birds, 100084 Beijing, China

**Keywords:** Climate and Earth system modelling, Climate-change mitigation

## Abstract

Bioenergy with carbon capture and storage (BECCS) is considered to be a key technology for removing carbon dioxide from the atmosphere. However, large-scale bioenergy crop cultivation results in land cover changes and activates biophysical effects on climate, with earth’s water recycling altered and energy budget re-adjusted. Here, we use a coupled atmosphere-land model with explicit representations of high-transpiration woody (i.e., eucalypt) and low-transpiration herbaceous (i.e., switchgrass) bioenergy crops to investigate the range of impact of large-scale rainfed bioenergy crop cultivation on the global water cycle and atmospheric water recycling. We find that global land precipitation increases under BECCS scenarios, due to enhanced evapotranspiration and inland moisture advection. Despite enhanced evapotranspiration, soil moisture decreases only slightly, due to increased precipitation and reduced runoff. Our results indicate that, at the global scale, the water consumption by bioenergy crop growth would be partially compensated by atmospheric feedbacks. Thus, to support more effective climate mitigation policies, a more comprehensive assessment, including the biophysical effects of bioenergy cultivation, is highly recommended.

## Abstract (web summary)

Increased global land precipitation, due to the atmospheric feedbacks of large-scale bioenergy cultivation, may partially compensate the water consumption by such rainfed bioenergy crops at the global scale.

## Introduction

As a consequence of the need to limit global warming below 2 or 1.5 °C in 2100^1^, attention is increasingly turning to negative emission technologies (NETs) as a way to counteract unreducible carbon emissions by removing carbon dioxide from the atmosphere^1–3^. With its dual function of carbon dioxide removal (CDR) and bioenergy supply, bioenergy with carbon capture and storage (BECCS) has been widely adopted in integrated assessment models (IAMs) as a means of achieving future temperature limiting targets^1^. Nevertheless, the CDR capacity of BECCS depends on factors other than just the biomass yield. For example, land-use change emissions^4^, losses in energy conversion^5^ and carbon capture^6^, and transport emissions^7^ reduce CDR of BECCS. Moreover, the feasibility of large-scale deployment of BECCS is also under debate due to uncertainty surrounding its ecological and economic effects. For instance, the water demand for supplying bioenergy crop irrigation is projected as 125–11,350 km^3^ year^−1^ (synthesized from 34 scenarios)^8^, which would intensify water stress worldwide^9,10^. However, reducing water use would increase the cultivation area required to produce the same amount of biomass^10^ provoking land-competition with food crops^11^. Monoculture and fast rotations of bioenergy crops may also exacerbate problems related to biodiversity loss^12^, nitrogen removal^13^, and soil erosion^14^, which would all involve additional economic costs^2^. The utilization of agricultural residues for BECCS has been proposed as one way to mitigate the adverse effects of bioenergy crop cultivation^15^, but the limited supply of such residues may not be sufficient for large-scale BECCS deployment in a timely manner^16^. In addition, large-scale bioenergy crop cultivation alters land surface properties and impacts the terrestrial energy and water balance through land–atmosphere interactions^2^. For example, a recent study has reported that the higher evapotranspiration rates and smaller aerodynamic resistances associated with bioenergy crop cultivations could result in a net cooling effect at the global scale compared with native vegetation. However, the impacts of large-scale bioenergy crop cultivation and associated land-atmosphere feedbacks on the terrestrial water cycle have yet to be systematically examined.

The terrestrial water balance evolves at different time scales and eventually reaches equilibrium through precipitation (*P*), evapotranspiration (ET), runoff, and water storage changes (WSC)^17^. Conversion of current land use to bioenergy crop cultivation impacts all these water balance components by altering surface roughness, stomatal conductance, and other land biophysical properties, with consequences that vary by region^18^. Insights gained from large-scale transformations of forest cover can provide hints about the unexplored impact of bioenergy crop cultivation. Large-scale deforestation in the Amazon has been shown to weaken the hydrological cycles both locally over the whole Amazon basin^19^ and over distant regions (e.g., Central America and the Gulf of Mexico)^20^, despite enhanced precipitation in the deforested patches^21^. Conversely, revegetation and afforestation lead to enhanced ET and precipitation^22^ and reduce the ratio of runoff to precipitation^23^.

The high biomass production of bioenergy crops relies on high biomass yields and frequent harvests^8^ of fast-growing species, and causes considerable water demands^24^. Previous studies^9,25–27^ have shown that additional water demand by bioenergy crops can exacerbate water scarcity even when sustainable water management schemes are in place. However, these assessments have not considered the potential feedbacks of vegetation changes on precipitation^9,10,25^. It remains unclear whether land–atmosphere feedbacks would exacerbate or alleviate the soil water deficits caused by enhanced transpiration^22^. Quantifying the biophysical effects of bioenergy crops on the terrestrial and atmospheric water cycle is thus an essential step towards demonstrating the feasibility of large-scale BECCS deployment and clarifying its limits for mitigating future climate change.

Here, a coupled land-atmosphere model (Institut Pierre-Simon-Laplace coupled model, IPSL-CM^28^) with explicit representations of bioenergy crops (e.g., eucalypt and switchgrass)^29^ has been used to simulate the responses of water exchanges between land and atmosphere to bioenergy crop cultivation (see Methods). Simulated biophysical properties and key hydrological variables have been extensively assessed and validated (Methods, Text S1 and Figs. S1–S3). A reference scenario without BECCS (S_ref_) and six BECCS scenarios (S_BECCS_) have been conducted for 50-year simulation periods (see Methods). S_ref_ uses the land cover map as observed in 2015, while S_BECCS_ has bioenergy crop cultivation in specified areas. Three bioenergy cultivation maps are considered. The first assumes that all marginal land becomes suitable for bioenergy crops (Campbell^30^), while the other two^31^ are based on land-use socio-economic models, with one assuming cultivation area is converted from forest (IMAGE^32^) and the other assuming cultivation area is converted from cropland, based on economic considerations (MAgPIE^33^) (Methods, Text S2 and Fig. S4). Six BECCS scenarios have been developed by separately considering two contrasting bioenergy crop types that broadly represent high-transpiration woody plants (i.e., eucalypt) and low-transpiration herbaceous (i.e., switchgrass) crops (Text S3). The effects of each BECCS scenario on the water cycle are determined by calculating differences in hydrological variables between the BECCS simulation and the reference simulation (S_BECCS_ − S_ref_).

## Effects of bioenergy crop cultivation on terrestrial precipitation

**Fig. 1:**
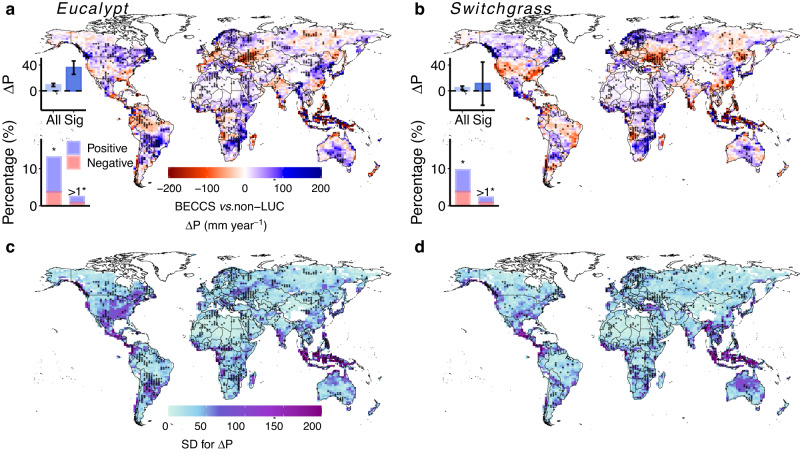
Spatial patterns of the changes in annual precipitation induced by eucalypt and switchgrass cultivation. Changes in annual precipitation (ΔP) are calculated as the differences of ten-year (i.e., the last ten years of the 50-year simulation period) annual precipitation between BECCS scenarios and the reference (no land-use change) simulation. **a**, **b** Spatial patterns of ΔP for eucalypt (**a**) and switchgrass (**b**) cultivation averaged over the three cultivation maps (Fig. S4). **c**, **d** Standard deviations (SD) of ΔP over the three cultivation scenarios for eucalypt (**c**) and switchgrass (**d**) cultivation. Stippling indicates that changes are statistically significant (according to the Wilcoxon signed-rank test at the 95% confidence level, *p* < 0.05) in at least one cultivation map. In (**a**, **b**), the upper insets show mean and standard deviations of ΔP over the entire global land area (left, labeled as “All”) and over regions with statistically significant precipitation changes (right, labeled as “Sig”). The lower insets show percentages of the global land area with significant changes detected in one (left) or more than one (right) of the three cultivation maps, with positive changes shaded blue and negative changes shaded red.

Changes in annual precipitation induced by large-scale bioenergy crop cultivation are shown in Fig. 1. Averaging across the three cultivation maps for each crop type, global-mean precipitation over land (excluding polar regions; see Methods) increases by 9.0 ± 2.4 mm year^−1^ (mean and standard deviation among the three cultivation maps) in the eucalypt scenarios and by 4.7 ± 2.7 mm year^−1^ in the switchgrass scenarios. For the eucalypt scenarios (Fig. 1a), significant (*p* < 0.05) precipitation changes (ΔP) are detected over 15.5% of the global land area, with an average ΔP of 35.4 ± 11.1 mm year^−1^. Precipitation increases in southern Canada, northern Europe, eastern China, central South America, southeastern Africa, and southern Australia (Fig. 1a). The switchgrass scenarios show similar spatial patterns of precipitation changes to the eucalypt scenarios (Fig. 1b), but with smaller magnitudes and spatial extents. For the switchgrass scenarios, significant precipitation changes (*p* < 0.05, ΔP = 11.1 ± 33.4 mm year^−1^) are detected over 11.9% of the global land area, mainly in northern Europe, northwestern Asia, southeastern Africa, and southern Australia (Fig. 1b).

Regional decreases in precipitation are found in both eucalypt and switchgrass cultivation scenarios, including in the southeastern U.S., southwestern Europe, Ukraine, northeastern and southern China, the Amazon basin, Angola, and southeastern Asia (Fig. 1). Notably, these regions include several important ecological conservation areas and agricultural zones, where decreased precipitation may further threaten local ecosystems^34^. Differences in ΔP between eucalypt and switchgrass are most pronounced in southern Europe, the northwestern and southeastern U.S. and southern Brazil (Fig. 1a, b), implying that precipitation in these regions is sensitive to bioenergy crop type. Precipitation changes also depend on the choice of cultivation map (Figs. 1c, d and S5), as ΔP varies substantially across the three cultivation maps in regions such as Central America and southeast Asia (large standard deviations in Fig. 1c, d). However, precipitation consistently increases in arid, semiarid, and subhumid zones (Methods and Fig. S6) for both bioenergy crop types and all three cultivation maps (Fig. S7), indicating that precipitation changes in these regions are robust to the choices of crop type and cultivation area.

## Diagnosing the precipitation changes

Physical processes behind precipitation changes (ΔP) can be diagnosed via moisture budget analysis^35^ (Methods and Text S4). The methodology is based on the budget equation of atmospheric water vapor^36^ and decomposes ΔP as changes in evapotranspiration (ΔET, locally provided), moisture convergence (Δ*Q*_cnvg_, locally transported, in relation to vertical motions), moisture advection (Δ*Q*_advt_, remotely transported) and a residual (Δ*ε*):

1 $$\Delta P=\Delta {{{{ET}}}}+\Delta {Q}_{{{{{{{\rm{cnvg}}}}}}}}+\Delta {Q}_{{{{{{{\rm{advt}}}}}}}}+\Delta \varepsilon$$

**Fig. 2:**
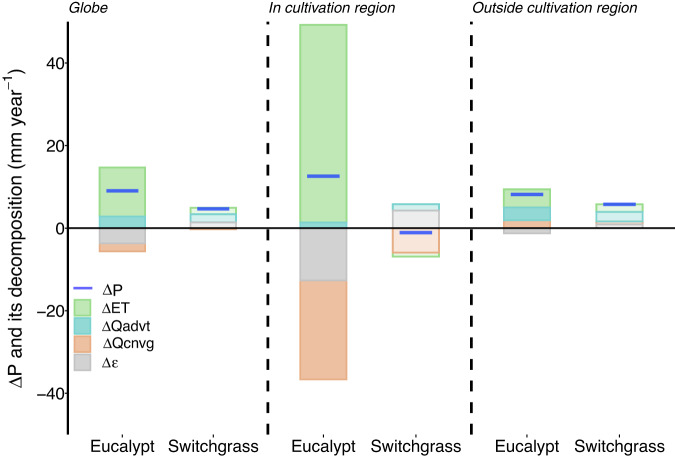
Diagnosis of global terrestrial precipitation changes. Stacked bars show the decomposing components of precipitation changes (ΔP) diagnosed from the atmospheric water vapor budget (Eq. (1)), including changes in evapotranspiration (ΔET), moisture advection (Δ*Q*_advt_), moisture convergence (Δ*Q*_cnvg_), and the residual term (Δ*ε*). Average changes across three cultivation maps for the eucalypt and switchgrass cultivation are aggregated for the entire global land area (left), the prescribed bioenergy crop cultivation regions (center) and the areas outside of the cultivation regions (right).

For the eucalypt scenarios, ΔET (11.9 ± 2.3 mm year^−1^) is the main term of increased moisture supply at global scale (Fig. 2). The leading role of ΔET is consistent both inside and outside the cultivation regions (Fig. 2) and across different humidity zones (Fig. S7). Positive contributions from ΔET (47.8 ± 13.5 mm year^−1^) are especially strong in eucalypt cultivation regions due to high rates of transpiration and photosynthetic production^8,24^, but much of this moisture supply is compensated by increased moisture divergence (negative Δ*Q*_cnvg_ of −24.0 ± 21.7 mm year^−1^). As a result, precipitation increases are comparable inside (12.6 ± 1.7 mm year^−1^) and outside (8.2 ± 3.4 mm year^−1^) eucalypt cultivation regions. For the switchgrass scenarios, ΔET (1.6 ± 1.2 mm year^−1^) and Δ*Q*_advt_ (2.0 ± 1.9 mm year^−1^) both contribute almost equally to the global increase in precipitation, while the compensating effect of Δ*Q*_cnvg_ is negligible (−0.3 ± 0.1 mm year^−1^). The increase in global precipitation under the switchgrass scenarios occurs mostly outside of bioenergy cultivation regions (5.8 ± 1.7 mm year^−1^), where contributions from ΔET, Δ*Q*_advt_, and Δ*Q*_cnvg_ are all positive (Fig. 2). In contrast, precipitation decreases locally in switchgrass cultivation regions owing mainly to large but variable negative contributions by Δ*Q*_cnvg_ (−5.9 ± 8.2 mm year^−1^; i.e., net moisture export to other regions). The diagnosed contributions to ΔP vary substantially across different humidity zones (Fig. S7). ΔET makes the largest contributions to ΔP in arid, semiarid, and humid zones, while Δ*Q*_advt_ plays the leading role in subhumid zones.

For both eucalypt and switchgrass, Δ*Q*_cnvg_ is negative over cultivation regions and positive outside them (Fig. 2), consistent with local biophysical cooling effects^37^ in the bioenergy crop cultivation regions and associated moisture divergence. Our results indicate that the export of local moisture from cultivation regions to other areas facilitates a more widespread influence of bioenergy crop cultivation on precipitation changes at global scale (Fig. 2). Values of Δ*Q*_advt_ are positive both inside and outside the cultivation regions (Fig. 2), suggesting that increases in low-level humidity and precipitation over land areas intensify the inland transport of water vapor from the ocean under the widespread bioenergy crop cultivation of our scenarios. Positive values of Δ*Q*_advt_ are found mainly over semiarid, subhumid, and humid zones, while average changes over the arid zone are negative (Fig. S7a), and consistent among the cultivation maps (Fig. S7b–d).

## Impacts on land water balance

**Fig. 3:**
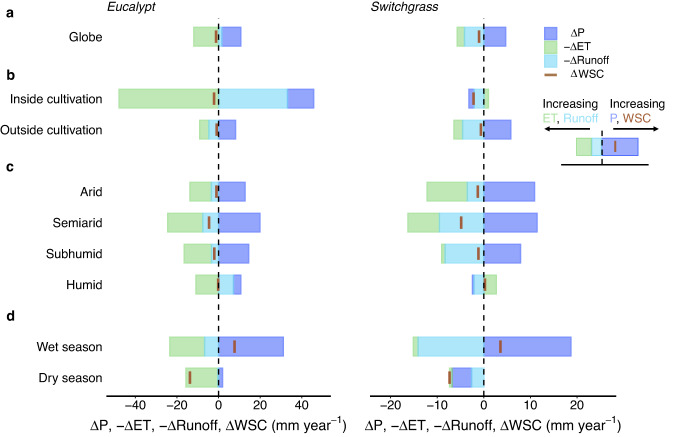
Changes in terrestrial water balance components at global and regional scales induced by bioenergy crop cultivation. **a** Changes in precipitation (ΔP), evapotranspiration (ΔET), runoff (ΔRunoff), and water storage changes (ΔWSC) at the global scale for eucalypt (left) and switchgrass (right) cultivation scenarios averaged over the three cultivation maps. Changes in water balance components are also aggregated for different regions: **b** inside and outside of the cultivation region, **c** various humidity zones (i.e., arid, semiarid, subhumid and humid zones), and **d** wet and dry seasons in the monsoon regions. Note that ΔET and ΔRunoff are shown with reversed signs (−ΔET and −ΔRunoff) to be consistent with the ΔWSC deduced from the water balance (Eq. 5), and the directions of changes in these items are illustrated in the stylized legend.

Further analyses explore the effects of bioenergy crop cultivation on individual components of the terrestrial water budget (see Methods), including water storage changes (ΔWSC), precipitation (ΔP), evapotranspiration (ΔET), and runoff (ΔRunoff). At the global scale, small reductions in WSC are found in both eucalypt (−1.2 ± 0.4 mm year^−1^) and switchgrass (−1.0 ± 0.7 mm year^−1^) scenarios, implying that bioenergy crop cultivation has a limited impact on soil water storage. The reasons for the small changes in WSC are, however, different for the two crop types (Fig. 3a). In the eucalypt scenario, the increased precipitation partly offsets the enhanced evapotranspiration (11.9 ± 2.3 mm year^−1^), leading to a slight reduction in soil water storage (Figs. 3a and S8a). The contribution of reduced runoff (−1.6 ± 1.5 mm year^−1^) to ΔWSC is relatively small. For the switchgrass scenarios, decreases in WSC result more from increased runoff than from increased ET (Figs. 3a and S9b). Negative ΔRunoff in eucalypt cultivation regions can be partially attributed to the water conservation effect of woody plants, such as precipitation interception by the tree canopy^38^. In contrast, interception by herbaceous plants is weak, and most of the increased precipitation would run off, explaining the positive ΔRunoff in the switchgrass cultivation regions (Fig. 3b). Overall, when results are averaged across the three cultivation-map scenarios, significant soil water reductions cover roughly 0.3% of the global land area in both eucalypt and switchgrass scenarios (Fig. S10).

Magnitudes of ΔWSC under the eucalypt and switchgrass scenarios are similar both inside (−2.2 ± 2.4 mm year^−1^ for eucalypt and −2.2 ± 3.2 mm year^−1^ for switchgrass) and outside (−0.9 ± 0.4 mm year^−1^ for eucalypt and −0.6 ± 0.1 mm year^−1^ for switchgrass) the bioenergy cultivation regions (Fig. 3b). However, other components of the land water balance differ substantially inside cultivation regions despite the broad consistency outside of these regions. In the eucalypt cultivation regions, the reduction in runoff (−33.1 ± 17.0 mm year^−1^) exceeds the increase in precipitation (12.6 ± 1.7 mm year^−1^), and thus plays the leading role in sustaining enhanced ET and balancing land water budgets. Yet, in the switchgrass cultivation regions, ΔP, ΔRunoff, and ΔET have similar magnitudes, with the net result of a small negative ΔWSC (Fig. 3b). Outside the cultivation regions, both for eucalypt and switchgrass scenarios, increased precipitation largely offsets increases in runoff and ET, leading to slight reductions in WSC. Although no land-use changes are prescribed outside of the bioenergy cultivation regions, atmospheric feedbacks, such as increased precipitation, intensify transpiration globally by supporting greater vegetation growth (as manifested by increased leaf area index, Fig. S11).

Regional analyses are also conducted for the changes in water balance among different humidity zones (see Methods). Arid, semiarid, and subhumid zones exhibit negative ΔWSC under both eucalypt and switchgrass scenarios owing to increased ET and runoff (Fig. 3c). When averaged across the humid zones, the ΔWSC is almost zero for both the eucalypt (−0.2 ± 0.6 mm year^−1^) and switchgrass (0.2 ± 0.7 mm year^−1^) scenarios. Meanwhile, changes in land water fluxes in the humid zones are different between the switchgrass and eucalypt scenarios. Runoff is reduced in the eucalypt scenarios, while the switchgrass scenarios show increased runoff and decreased ET (Fig. 3c). These results suggest that the feedbacks on the terrestrial water balance are more dependent on the cultivation area and crop type than on the local humidity.

As the near-zero ΔWSC in humid areas may conceal compensating seasonal variations, we further examine water balance changes during the wet and dry seasons in monsoon-affected areas (Text S5 and Fig. S12). During the wet season, increased precipitation completely offsets increased runoff and ET, resulting in a slight positive ΔWSC under both scenarios (Fig. 3d). In contrast, during the dry season, ΔWSC in monsoon areas is negative, at both global (−13.8 ± 5.1 and −7.4 ± 6.7 mm year^−1^ for eucalypt and switchgrass, respectively) and regional scales (Fig. S13). The soil water deficits during the dry season mainly result from enhanced ET in the eucalypt scenarios and from decreased precipitation in the switchgrass scenarios (Figs. 3d and S13). Wetter soil during the wet season and drier soil during the dry season suggest that bioenergy crop cultivation could aggravate the tendency toward “wetter wet season and drier dry season” that has been both observed and predicted for monsoon regions under climatic warming^39–42^. This tendency has also been linked to extreme events (e.g., drought and pest outbreaks) and the consequential ecological effects (e.g., tree mortality)^43,44^. These aspects need to be properly evaluated and considered to support bioenergy policy development and implementation.

## Discussion

Our simulations demonstrate that land-atmosphere feedbacks associated with large-scale bioenergy crop cultivation increase precipitation over land, and that this increase can largely alleviate the enhanced soil water stress brought about by large-scale bioenergy crop cultivation. We estimate that, when atmospheric feedbacks are considered, only 0.3% of the global land area in S_BECCS_ will experience a significant soil water reduction, compared to S_ref_. Previous studies have estimated increased water stress due to bioenergy crop irrigation^9^ and the large water consumption for bioenergy crops would cause water shortages for traditional agricultural use^10^. Considering the steep costs and ecological impacts of large-scale irrigation^45^, we assume that all bioenergy crops are rainfed in our scenarios. The increased precipitation outside of bioenergy crop cultivation regions, due to the climate feedbacks, may further reduce the water demands of agricultural irrigation dedicated to human food and animal feed. On the other hand, the reduced runoff may decrease the freshwater supply to reservoirs. However, the hydrological changes show strong spatial variations (Figs. 1 and S8–S10), and the impacts on irrigation thus need further investigation.

Changes in the water balance are generally consistent for the eucalypt and switchgrass scenarios outside of the cultivation regions, while differences within the cultivation regions (Fig. 3b) imply distinct local atmospheric feedbacks for woody versus herbaceous crops. Eucalypt has larger transpiration and canopy interception rates than switchgrass^24^, which accelerates the water cycle around eucalypt plantations relative to that around switchgrass sites. These different cycling rates also apply in comparison to the original vegetation without bioenergy crop cultivation. For example, global-scale precipitation increases under both the Campbell and IMAGE eucalypt scenarios (Fig. S5a, b), while the global-mean precipitation changes are positive in the Campbell switchgrass scenarios (Fig. S5d) but negligible in the IMAGE switchgrass scenarios (Fig. S5e). Most of the cultivation areas on the Campbell map are converted from short vegetation (cropland, grassland, and pasture), while 78% of the source land areas on the IMAGE map are forested (Fig. S4). The different precipitation responses to large-scale switchgrass cultivation between these two scenarios thus highlight that appropriate land selection for conversion to bioenergy crop cultivation can limit the additional strain placed on water resources.

The switchgrass scenarios indicate increases in precipitation over many temperate areas with large river basins (Fig. 1), which could heighten the risk of flood events^46,47^. Likewise, increased water demand associated with bioenergy crop cultivation in arid and semiarid zones (Fig. 3c) may exacerbate local water stress and reduce ecosystem resilience. Decreases in precipitation are simulated for several biodiversity hotspots with high ecosystem productivity, including the Amazon basin and southeast Asia^48^. Reduced rainfall in these regions may intensify species loss^34^, tree mortality^49^, and local carbon emissions^50,51^, thereby raising the environmental cost and reducing the carbon removal efficiency of BECCS projects. Reductions in precipitation are also simulated for several important agricultural regions in Europe, such as Ukraine, Germany, and the Netherlands, possibly lowering yields. The likelihood, ecological impacts, and associated socio-economic costs of these possible adverse effects of BECCS must be thoroughly evaluated. For example, the possible increases in flood risk could be assessed by coupling with flood models^47,52^, and the associated economic losses could be further calculated by using flood depth-damage functions^53^.

Although small-scale bioenergy crop cultivation is also an important component of BECCS implementation, it is not considered in our study due to the coarse model resolution (see Methods). Small-scale bioenergy cultivation can alter local energy balance and moisture circulations, and the impacts of associated atmosphere feedbacks on regional hydrological balance may be limited^54,55^. The biophysical effects of large-scale cultivation might differ from those of scattered cultivation, and should not be downscaled, given the complexity and nonlinearity of climate feedbacks. Nevertheless, the diagnostic framework for separating the impacts of local and non-local provided moisture on precipitation changes establishes a foundation for future research on exploring the locally hydrological effect of bioenergy crop cultivation and optimizing the most suitable crops under mosaic cultivation scenarios in given regions (Text S6). Until now, a few other land surface models (e.g., LPJmL^56^, JULES^57^, and CLM^58^) have implemented bioenergy crop modeling, and these models may also have the capacity for simulating the hydrological processes of bioenergy crops and evaluating the land-atmosphere feedbacks by coupling with a general circulation model. Future coupled model intercomparison projects designed for BECCS are expected to provide more robust results for the climate feedbacks of bioenergy crop cultivation.

The results of this study highlight the fundamental ways in which large-scale BECCS deployment could alter the terrestrial water cycle. The feasibility of BECCS depends not only on its CDR potential but also on biophysical constraints such as water availability and changes in the local atmospheric temperature and humidity. A comprehensive assessment of both biogeochemical and biophysical impacts of BECCS is therefore an essential step toward developing strategies and policies that better mitigate the effects of anthropogenic climate change.

## Methods

### Simulation design

IPSL-CM has been used to simulate the coupled global atmosphere and land surface system, components which are separately simulated by the global atmospheric model, LMDZ6^59^, and the global dynamic vegetation model, ORCHIDEE-MICT-BIOENERGY^29^. Sea surface temperature (SST) and sea-ice conditions were prescribed as fixed external forcings with climatological annual cycles derived from the Atmospheric Model Intercomparison Project (AMIP, http://www-pcmdi.llnl.gov/projects/amip). LMDZ6 is the latest version of the Laboratoire de Météorologie Dynamique atmospheric general circulation model, and has been used to conduct numerous simulations for CIMP6^28^. It simulates all fundamental dynamic and physical processes on a regular grid of 2.5 degrees in longitude by 1.25 degrees in latitude with 79 vertical layers^22,28^. Land-surface processes are interactively coupled at 30-min intervals by using the ORCHIDEE-MICT-BIOENERGY, which is programmed to represent biomass yields of lignocellulosic bioenergy crops such as eucalypt and switchgrass^29^. Parameters constraining ecological processes (e.g., photosynthesis, carbon allocation, phenology and biomass harvest) for bioenergy crops have been systematically calibrated against field measurements^29^, and the model performance has been validated by previous studies^22,29,60^. The model has been shown to reliably reproduce the yield, albedo, and evapotranspiration of bioenergy crops^29^. Further validation of the simulated hydrological variables in this study is provided in Text S1.

Using this coupled model, six BECCS scenarios (2 bioenergy crop types × 3 cultivation maps) and one reference scenario were conducted. For each scenario, a fifty-year simulation was carried out. Eucalypt and switchgrass were selected to represent high-transpiration woody and low-transpiration herbaceous bioenergy crops, respectively (Text S3). The harvest cycle for eucalypt was set to every five years. Three bioenergy crop cultivation maps were used (Text S2 and Fig. S4). (1) The first map (Campbell) specifies abandoned agricultural lands as bioenergy cultivation areas, as proposed by Campbell et al.^30^. These areas are widely distributed across the globe and predominantly covered by short vegetation. (2) The second map is derived from a land-use socio-economic model, IMAGE^32^. It assumed that most cultivation areas are converted from forests and very few from croplands, to avoid land competition between bioenergy crops and food crops. (3) The third map is obtained from another socio-economic model MAgPIE^33^, which assumes that cultivation land is mainly converted from cropland. In MAgPIE, land competition between bioenergy crops and food crops is resolved based on cost minimization. For the reference scenario, the land cover map for 2015 is adopted, with all the other settings being the same as in the BECCS scenarios.

### Region division

The cultivated area is specified as grid cell fractions according to the three cultivation maps. The total global area for bioenergy crop cultivation ranges from 432 to 578 M ha across these three maps (Fig. S4). Because the cultivation area in the Campbell map is derived from abandoned land that is widely distributed across the globe, the fraction in each grid cell is relatively low (Figs. S4 and S14a). In contrast, the cultivation areas in the IMAGE and MAgPIE maps are more concentrated in a few regions (e.g., Europe, central North America and central Africa) and is generally specified by large cultivation fractions in the affected grid cells.

Because most of the hydrological variables simulated by the model are for the entire grid cell (i.e., not specified according to plant functional types), a threshold cultivation fraction (*f*_Bcrop_) is needed to select grid cells for the bioenergy cultivation regions. We tested a range of threshold bioenergy crop fractions (*f*_Bcrop_ = 0.1, 0.08, 0.05, 0.01, 0.005) to define the cultivation region (Fig. S14). The value *f*_Bcrop_ = 0.05 was selected as the default value, as a compromise between the low fractions in the Campbell map and the narrow distributions in the IMAGE and MAgPIE maps (Figs. S4 and S14). Using this threshold, the specified cultivation regions account for 74%, 94%, and 95% of the total cultivation area in the Campbell, IMAGE, and MAgPIE maps, respectively. The different values of *f*_Bcrop_ have little impact on the water balance changes or precipitation diagnostics calculated for cultivation regions (Fig. S15).

We used the aridity index (AI) dataset from the Food and Agriculture Organization (FAO) Statistics^61^, combined with the recently updated Köppen-Geiger classification (present day, 1980–2016)^62^ to classify global land areas into arid, semiarid, subhumid, humid and polar zones (Fig. S6). The FAO aridity index data is the ratio of multi-year (1961–1990) average precipitation divided by potential evapotranspiration (PET) based on Climate Research Unit (CRU) climate data using the Penman-Monteith method^61^. The aridity index from FAO, which ranges from 0 to 10.48, is reported for each 10-arc-minute grid cell. In this study, the AI data were first aggregated to a spatial resolution of 1.26° latitude × 2.5° longitude using the bilinear interpolation method. Global land grid cells were then classified into arid (AI < 0.2), semi-arid (0.2 ≤ AI < 0.5), subhumid (0.5 ≤ AI < 0.65), and humid (AI ≥ 0.65) zones^61,63^. Grid cells that are defined as climate type E (polar climate) in the Köppen-Geiger classification system, were cataloged as polar zone, and excluded from our analysis (Fig. S6 and Table S1).

The monsoon area is defined based on the monsoon precipitation index (MPI) and the annual range (AR) of precipitation between wet and dry seasons^64,65^. Following Wang and Ding^64^, land areas where MPI > 0.5 and AR > 300 mm are identified as monsoon areas (Text S5). The six identified monsoon regions correspond to the North American, South American, North African, South African, Asian, and Australian monsoons (Fig. S12 and Text S5).

### Diagnosis of precipitation changes

The atmospheric water vapor budget equation^36^ has been widely used in diagnosing the changes in precipitation by splitting vertically integrated moisture-flux divergence into two terms, namely, moisture convergence (*Q*_cnvg_) and moisture advection (*Q*_advt_)^35,66,67^:

2 $$\frac{\partial {W}}{\partial {t}}-(\underbrace{(-W\nabla \cdot \overrightarrow{V})}_{{{Q}_{{{{{{\mathrm{cnvg}}}}}}}}}+\underbrace{(-\overrightarrow{V}\cdot \nabla W)}_{{{Q}_{{{{{{\mathrm{advt}}}}}}}}})={{{ET}}}-P$$

where *W*, *P*, and ET are total water vapor content, precipitation, and evapotranspiration, respectively, and the vector $\vec{V}$ indicates atmospheric wind (see Text S4). *Q*_cnvg_ and *Q*_advt_ are measurements of atmospheric circulation convergence and horizontal moisture inhomogeneity^68^. The tendency term $\frac{\partial W}{\partial t}$, which is the rate of change of column-integrated atmospheric moisture, can be regarded as zero if the system is in a steady state^35^. Moving the subtracted P from right to left, Eq. (2) can be rearranged to give P as:

3 $$P={{{ET}}}+(\underbrace{(-W\nabla \cdot \overrightarrow{V})}_{{Q}_{{{{{\rm{cnvg}}}}}}}+\underbrace{(-\overrightarrow{V}\cdot \nabla W)}_{{Q}_{{{{{\rm{advt}}}}}}})+\varepsilon$$

The *ε*, is a residual term representing the co-variate effect between *W* and $\vec{V}$, and it can be omitted when mean values of each variable are used in Eq. (3). It also includes unavoidable numerical errors in calculating both wind divergence and water vapor gradient (small amount issued from the subtraction of two large amounts). Thus, the precipitation changes (ΔP) induced by bioenergy crop cultivation are diagnostically attributed to the respective contributions of ΔET, Δ*Q*_cnvg_, and Δ*Q*_advt_ (Eq. (1), Δ denotes the difference between S_BECCS_ and S_ref_ for a given variable). Model simulated specific humidity (*q*), air pressure (*p*), and meridional and zonal windspeeds were used to calculate *Q*_cnvg_ and *Q*_advt_ (see Text S4).

### Analysis

In the coupled simulations, key vegetation features (e.g., photosynthesis and leaf area index) related to the energy budget (e.g., evapotranspiration and albedo), reach dynamic equilibrium in approximately 10 years^37^. We therefore treat the first 10 years of each simulation as the spin-up period and analyze only the last 10 years. The global increase in precipitation is robust to the choice of analysis periods (e.g., the last 20 years, Fig. S16). Statistical significance for each estimation is evaluated by using the Wilcoxon signed-rank test. Global and regional averages are derived as area-weighted means. All the analyses were conducted using R 4.1.3 (https://www.r-project.org/).

The terrestrial water fluxes consist of (1) precipitation (P), comprising both rainfall and snowfall; (2) evapotranspiration (ET), comprising land surface (soil and canopy) evaporation, vegetation transpiration and snow sublimation; and (3) runoff (surface or subsurface streamflow). Changes in these fluxes result in (4) water storage changes (WSC) in land surface pools (e.g., snow-pack, vegetation, lakes, wetlands, rivers, reservoirs) and subsurface pools (e.g., soil moisture and groundwater)^17,69^. With these four components, the terrestrial water budget, based on a mass balance, can be closed and expressed as:

4 $${{{WSC}}}={{{P}}}-{{{ET}}}-{{{Runoff}}}$$

In our study, WSC is calculated as the residuals of the right-hand side of Eq. (4) for each simulation in each grid cell. The impacts of bioenergy crop cultivation on the terrestrial water balance are computed by replacing each component in Eq. (4) with the difference in its value between the BECCS simulations and the reference simulation (S_BECCS_ − S_ref_) i.e.:

5 $$\Delta {{{WSC}}}=\Delta {{{P}}}-\Delta {{{ET}}}-\Delta {{{Runoff}}}$$

## Supplementary information

Supplementary Information

## Data Availability

The data that support the main findings of this study and the corresponding coding scripts have been provided through the following URL https://zenodo.org/record/8041494. Datasets used for model validation are publicly available through CRU^70^ (https://crudata.uea.ac.uk/cru/data/hrg), GPCC^71^ (https://opendata.dwd.de/climate_environment/GPCC), FLUXCOM^72^ (https://www.bgc-jena.mpg.de/geodb/projects/FileDetails.php), GLEAM^73^ (https://www.gleam.eu/), and G-RUN^74^ (https://figshare.com/articles/dataset/G-RUN_ENSEMBLE/12794075).

## Code availability

The source code of ORCHIDEE-MICT-BIOENERGY is publicly accessible through 10.14768/02v2-z742. Information about the ORCHIDEE land surface model and ongoing developments could be found at the project homepage, https://orchidee.ipsl.fr/. ORCHIDEE-MICT-BIOENERGY is governed by the CeCILL licence under French law and is bound by the rules of free software distribution. One can use, modify, and/or redistribute the software under the terms of the CeCILL licence as circulated by CEA, CNRS, and INRIA via http://www.cecill.info.
