# Supplementary material for: Increased precipitation over land due to climate feedback of large-scale bioenergy cultivation
Source: Nat Commun. 2023 Jul 11;14:4096. doi: 10.1038/s41467-023-39803-9 (PMC10336109; doi:10.1038/s41467-023-39803-9)
Supplement: Supplementary file 1 — Supplementary Information [file 41467_2023_39803_MOESM1_ESM.pdf]

Increased precipitation over land due to climate feedback of large-scale bioenergy cultivation

Supplementary Information

Supplementary Text

Text S1. Model valuation

S1.1 Validation in previous studies

The IPSL-CM used in this study is a coupling of LMDZ6[1] and ORCHIDEE-MICT-BIOENERGY[2]. LMDz6 is the latest atmospheric general circulation model to be used in IPSL-CM. Compared to the previous version, (LMDZ5B: the "New Physics" version)[3], LMDZ6 includes modifications to the thermal plume model, the radiation scheme and the cloud-water-content algorithm[1], and can better simulate climate variations (e.g., radiation, temperature, precipitation and wind) than previous versions[4]. It should be noted that the representation by LMDZ of the transpiration-evapotranspiration proportion, surface radiation and surface energy fluxes has been validated against stable-isotope-based estimates, satellite observations and reanalysis datasets[5,6]. ORCHIDEE-MICT-BIOENERGY is an extended version of the land surface model, ORCHIDEE, in which biogeochemical processes, e.g., the terrestrial water balance and the carbon cycle, have been fully validated by site-level and global-scale observations[7-9]. In particular, for the plant function types (PFTs) of bioenergy crops, the ORCHIDEE-MICT-BIOENERGY has been calibrated by using 136 site-year measurements of plant growth dynamics and a global observational dataset of biomass yield for four bioenergy crops[2].

IPSL-CM represents the response of evapotranspiration[10] and surface energy fluxes[5] to LAI changes in natural ecosystems well[10]. Simulated temperature changes (due to both the local energy budget and the regional atmospheric circulation) and hydrological changes[10] have been shown to be comparable and consistent with other models, satellite observations and reanalysis climate data[11]. In addition, this coupled model version (LMDZ6 and ORCHIDEE-MICT-BIOENERGY) has been used to simulate some idealized bioenergy crop cultivation scenarios. The simulated seasonal variation and annual magnitude of albedo and evapotranspiration have been reported to

show a good performance against field measurements from 19 FLUXNET crop sites and >40 bioenergy crop sites[12].

S1.2 Validation for hydrological variables

Observation-based precipitation (i.e., CRU TS4.05[13] and GPCC[14]) and ET (FLUXCOM[15] and GLEAM[16]) datasets were used to validate the model's representation of annual mean precipitation and ET. The Gridded Climatic Research Unit (CRU) Time-series (TS) dataset is a widely used observation-based monthly dataset, provided by the UK's National Centre for Atmospheric Science (NCAS) on a spatial grid of 0.5° in both latitude and longitude. The CRU dataset was derived from daily or sub-daily observations collected from 2,600 stations worldwide and interpolated onto the regular grid with the Angular-distance weighting (ADW) method[17]. Version 4.05 (CRU TS4.05)[13], used in our model validation, is an improved dataset with a revised interpolation function and covers the period 1901-2020.

Global land-surface precipitation data products from the Global Precipitation Climatology Centre (GPCC) are rain-gauge-based global gridded precipitation databases[18]. With long-term *in-situ* measurements from more than 85,000 rain-gauge stations worldwide, the GPCC provides four precipitation products (i.e. Precipitation Climatology, CLIM; Full Data Reanalysis Product, FD; Monitoring Product, MP; and the First Guess Product, FG) with spatial resolution specifications of 0.25° × 0.25°, 0.5° × 0.5°, 1.0° × 1.0° and 2.5° × 2.5°, respectively. The Full Data Monthly Product V.2020 (for 0.5° resolution)[14], which has higher accuracy and is recommended for hydro-meteorological model verification, was used in our model validation.

The FLUXCOM energy flux product is a machine-learning-method based global gridded dataset, which upscaled site-level energy fluxes from eddy covariance towers using satellite and meteorological observations[15,19]. There are two setups used to produce the dataset, one exclusively involving MODIS remote sensing data (RS), and the other additionally including meteorological data (RS+METEO)[15]. For the two setups, nine and three machine learning algorithms were trained separately. Before model training, the energy-flux measurements were first corrected using the energy balance, generating three corrected variants of the latent (LE) and sensible (H) heat fluxes. Therefore, 27 (9 × 3) products of LE/H were yielded by the RS setup and 36 (12 × 3) products (four climate datasets put in RS+METEO) of LE/H were produced by the

RS+METEO setup. To capture the uncertainties among the machine learning methods or climate datasets, the ensemble products, estimated as the median energy flux over all ensemble members in each grid cell and month are provided for each setup. With their better representation of local-scale heterogeneities and lower uncertainties among the ensemble methods, the RS products are more suitable for validating land surface models[15]. The monthly latent heat flux values from the RS ensemble (0.5° grid cells) with a latent heat of vaporization of 2.45 MJ mm^{-1} were used in evapotranspiration conversion and model validation.

The Global Land Evaporation Amsterdam Model (GLEAM) was designed to estimate terrestrial evaporation and root-zone soil moisture with satellite-derived data[16,20]. The set of algorithms has been improved in the third version (v3) by modifying the representation of evaporative stress and the water balance, as well as the strategy for soil moisture data assimilation[16]. The GLEAM v3 produced three datasets for both evapotranspiration and soil moisture, separately using satellite-observed forcing alone (v3b and v3c) or accompanied by climate variables (v3a) as inputs. The evaporation estimations of the three datasets are very similar, while a comparison with the measurements from 2,325 sites, shows the soil moisture in v3a is more accurate than in v3b and v3c[16]. For each dataset, version 3 has higher accuracy than version 2 data compared with the in situ measurements[16]. Yearly evapotranspiration data with a spatial resolution of 0.25° × 0.25° from GLEAM v3a was used in the model validation here.

All the observation data were first resampled to 1.26° latitude × 2.5° longitude and then aggregated to yearly sums. The annual mean values of P and ET during 2005-2015 obtained from observation datasets were used to validate the yearly P and ET averaged from 2055-2064 from the reference simulation (S_{ref}) with the present land covers. As shown in Fig. S1, our model can generally capture the spatial patterns of P and ET with latitude and across biomes. Regionally, annual P is underestimated by the model in the southern Amazon basin and overestimated in some regions such as northeast Asia. For ET, the two observation-based datasets show substantial differences at the regional scale (Fig S1 e, f), and our model results are generally within the range of observations (Fig. S1d). The model simulates higher ET in northern Siberia but lower ET in the tropics when compared to FLUXCOM, and lower ET in northern Siberia and higher ET in the tropics compared with GLEAM v3a. Using values from all the grid cells, the scatter plots (Fig. S2) show strong correlations between observed and simulated values

both for P ($r = 0.7$, $p < 0.01$ for CRU; and $r = 0.7$, $p < 0.01$ for GPCC) and ET ($r = 0.9$, $p < 0.01$ for FLUXCOM; $r = 0.8$, $p < 0.01$ for GLEAM).

In addition, we also validated the model performance on total runoff using an observation-based global runoff reanalysis gridded dataset, Global RUNoff ENSEMBLE (G-RUN ENSEMBLE[21]). The G-RUN ENSEMBLE is an upscaled machine-learning dataset trained by global streamflow observations over the period 1902–2019 (Global Streamflow Indices and Metadata Archive, GSIM[22,23]) and forced by multiple climate datasets. The estimates of the global mean runoff volume of the 525 members in the G-RUN ENSEMBLE range between 3.2×10^4 and 3.8×10^4 km^3 yr^{-1}, a range which is comparable with the estimate from 25 state-of-the-art global hydrological models of $3.0 \times 10^4 \sim 4.1 \times 10^4$ km^3 yr^{-1} (e.g., ensemble median of ISIMIP2a[24]). The multi-model median of all the 525 members (G-RUN ENSEMBLE MMM) was used to validate our model.

Similarly to the validation of precipitation and ET, the G-RUN ENSEMBLE runoff data was resampled to 1.26° latitude × 2.5° longitude grids, and then aggregated over the period 2005-2015 to multiple-year averages to compare with the yearly runoff of the last 10 simulation years from the reference simulation (S_{ref}). As shown in Fig. S3, our model can capture the spatial patterns and the magnitudes of the observation-based runoff. Across all grid cells, significant correlations between observation-based (G-RUN ENSEMBLE) and the simulated Runoff ($r = 0.6$, $p < 0.01$) were detected (Fig. S3c). At the regional scale, the annual runoff is overestimated in eastern Siberia, and northeast Oceania, and underestimated in some regions such as northeast Asia (Fig. S3a, b).

Overall, the IPSL-CM can generally reproduce the main hydrological variables in the terrestrial water balance.

Text S2. Bioenergy crop cultivation distribution

To minimize competition for land with food crops and forests, marginal land is one of the proposed land types for bioenergy crop cultivation[25]. Therefore, we used an estimate based on global marginal land assumed to be available for bioenergy cultivation by Campbell et al. (2008)[26] (Campbell), as one cultivation map in our BECCS scenarios. For Campbell, marginal land was defined as the total area of abandoned cropland and abandoned pastures. In each grid cell, the difference between

the maximum historical cropland/pasture area since 1700 and the cropland/pasture area in 2000 was taken as the abandoned area. The History Database of the Global Environment, HYDE 3.0[27] and MODIS Land Cover Type Product[28] were separately used to obtain the historical and present-day land cover areas. Note that the transition from agricultural land to urban development or forest was excluded.

We also used two other cultivation maps obtained from two different IAMs, IMAGE[29] and MAgPIE[30], both of which deploy BECCS based on Representative Concentration Pathway (RCP) 2.6 and Shared Socio-economic Pathway (SSP) 2 scenarios[31,32]. In these two land-use scenarios, BECCS was assumed to be the only land-based mitigation option employed to achieve the CO_2 removal target. The historical patterns of cropland and pasture in both IAMs were harmonized with the HYDE 3.1 dataset[33], and the future land-use change predictions started in 2005. The land allocation rules were different for IMAGE and MAgPIE. In IMAGE, only non-food production regions were allowed to cultivate bioenergy crops, while in MAgPIE, bioenergy crop cultivation can compete for fertile land with food crops, based on cost minimization[34].

The global total area for bioenergy crop cultivation is 497.6 M ha in the Campbell map (Fig S4a, d), and the land sources are pasture (39%), grassland (35%) and cropland (26%). In the IMAGE and MAgPIE maps, the total bioenergy crop cultivation areas are 577.7 M ha and 432.2 M ha respectively, with 78% of the land converted from forest in IMAGE and 75% from cropland in MAgPIE.

Text S3. Bioenergy crop types

We chose eucalypt and switchgrass as representative woody and herbaceous bioenergy crops because of their distinct biophysical properties (e.g., albedo, evapotranspiration and LAI)[12]. Also, based on the gridded yield dataset upscaled by a machine-learning method[35], they represent a higher and lower range of yields in most regions. We acknowledge, however, that upscaling scattered field observations to a large scale has limitations due to there being few field data in some regions. For example, Europe and North America have few eucalypt observations, and there is barely any data available for poplar, willow, miscanthus and switchgrass in the tropics[2]. More field measurements for bioenergy crops are thus needed, in particular in underrepresented regions. However, model simulations with a more realistic mixture of different bioenergy crops in each region would complicate the isolation of the signal of

biophysical feedback compared to our tailored simulations with exclusive cultivation of eucalypt and switchgrass.

Text S4. Diagnosing precipitation changes

The atmospheric water vapor balance equation has already been reported by Starr and Peixoto (1958)[36,37], as a powerful tool for understanding the water vapor budget in the atmosphere. With the assumption of hydrostatic equilibrium, the atmospheric water vapor balance for a column of air can be expressed (Rasmusson, 1968)[38] as:

$$\frac{\partial W}{\partial t} + \nabla \cdot Q = \sum \tag{6}$$

Where, W is the column-integrated precipitable water, given by the vertically integrated specific humidity across pressure level ($W = \int_{P_{top}}^{P_{sruf}} q \frac{dp}{g}$). Q is the vertical integrated atmospheric moisture flux, a product of wind and specific humidity[39]:

$$Q = \int_{P_{top}}^{P_{sruf}} (\vec{u}q) \frac{dp}{g} \tag{7}$$

Weighted by W and W^{-1}, Equation (6) can be further transformed as:

$$\frac{\partial W}{\partial t} + \nabla \cdot \left(W\vec{V}\right) = \sum \tag{8}$$

Where $\vec{V}$ is the total horizontal moisture transport normalized by the column-integrated moisture:

$$\vec{V} = \frac{1}{W} \int_{P_{top}}^{P_{sruf}} (\vec{u}q) \frac{dp}{g} \tag{9}$$

Evapotranspiration (ET), the water flux from the earth's surface to the air column, and precipitation (P), falling from the air water vapor columns, are the explicit source and sink of atmospheric water vapor. Thus, $\sum = ET - P$, and Equation (6) and (8) can be combined to give[40,41]:

$$\frac{\partial W}{\partial t} + \nabla \cdot \left(W\vec{V}\right) = ET - P \tag{10}$$

According to the divergence algorithm, $\nabla \cdot \left(W\vec{V}\right)$ is equal to the sum of two terms $\left(W\nabla \cdot \vec{V}\right) + (\vec{V} \cdot \nabla W)$. The former term $(W\nabla \cdot \vec{V})$ represents the dynamical convergence effect of the atmospheric circulation ($-Q_{cnvg}$), while the later term, $(\vec{V} \cdot \nabla W)$ is the

moisture advection ($-Q_{advt}$). Equations (6), (8) and (10) provide the basis for Equations (2) and (3) in the main text.

Q_{cnvg} and Q_{advt} measure the effects of atmospheric circulation convergence and that related to the horizontal moisture inhomogeneity[40], respectively. For each month-grid and simulation, Q_{cnvg} and Q_{advt} were calculated based on model simulated specific humidity (q), air pressure (p) and the meridional and zonal wind components. Related calculations were conducted with the NCAR Command Language (NCL) http://www.ncl.ucar.edu/.

Text S5. Identification of monsoon areas

Monsoon is a response of the coupled atmosphere-land-ocean system to seasonal variations in the solar radiation forcing, and dominates the seasonal variations of tropical precipitation[42]. The monsoon region is distinguished from other regimes by its prominent characteristics of high annual totals and high seasonality of precipitation[42,43]. Thus, precipitation is a fundamental variable for the investigation of the monsoon climate regime. Previous studies have explored a series of precipitation indexes or criteria to define the monsoon regions[43-46]. Here, we adopted the method of Wang and Ding (2008)[43] and used the annual precipitation range (AR) and monsoon precipitation index (MPI) to define the monsoon regions.

AR is the difference in the total precipitation between local summer and winter. MPI is the ratio between AR and the annual total precipitation:

$$MPI = \frac{AR}{Total\ annual\ precipitation} \tag{11}$$

For land in the Northern Hemisphere, the wet and dry seasons are defined as the period from May to September (MJJAS) and from November to March (NDJFM), respectively. For the Southern Hemisphere land, the wet and dry seasons are defined as November to the next March (NDJFM) and from May to September (MJJAS), respectively.

The monthly precipitation (averaged from 1970~2000) obtained from WorldClim V2 (https://worldclim.org/data/worldclim21.html) is used to calculate AR and MPI. Following Wang and Ding (2008)[43], land with MPI > 0.5 and AR > 300 mm are identified as monsoon areas. As a result, six monsoon regions are identified: the North American (N_Am), South American (S_Am), North African (N_Af), South African

(S_Af), Asian (As), and Australian (Au) monsoon regions (Fig S12). Note that the Tibetan Plateau was excluded from our analysis, as the polar zone was not included in our analysis.

Text S6. Most suitable bioenergy crop type

The climate feedbacks on precipitation in a specific region are impacted by both changes in local conditions and land-use changes in other regions (through atmospheric circulation). Therefore, from a global perspective, it is difficult to define crop types that alleviate regional water stress, as they affect climate in other regions and are affected by crops cultivated elsewhere. Yet, we try to isolate the contributions of crop types to the precipitation change in the region in which they are cultivated (ΔP_{local}) based on the water budget (Eq. 1). Q_{advt} is the precipitable water remotely transported by atmosphere circulations, which also depends on land-use changes in other regions. We assume that changes in precipitation induced by changes in ET and Q_{cnvg} are local (i.e., ΔP_{local} = $\Delta ET + \Delta Q_{cnvg}$), and then compare the regional ΔP_{local} between the eucalypt and switchgrass scenarios to see which crop could bring more "local" precipitation (Table S2, Fig. S17).

We find that eucalypt cultivation has a positive influence on local precipitation in most regions except southeast Asia. The impacts of switchgrass cultivation on local precipitation vary across regions. In the switchgrass scenarios, positive precipitation changes are detected in Africa, Pacific developed regions, South and Central America, and the Middle East, while negative changes are found in south, southeast, and east Asia. In regions with positive ΔP_{local} for both switchgrass and eucalypt cultivation, such as Eurasia, the Pacific developed region, and Africa, the magnitude of ΔP_{local} is still smaller in the switchgrass scenarios than in the eucalypt scenarios. Thus, regions would generally gain more local precipitation with eucalypt cultivation than with switchgrass cultivation. Nevertheless, more precipitation would also increase the risk of floods.

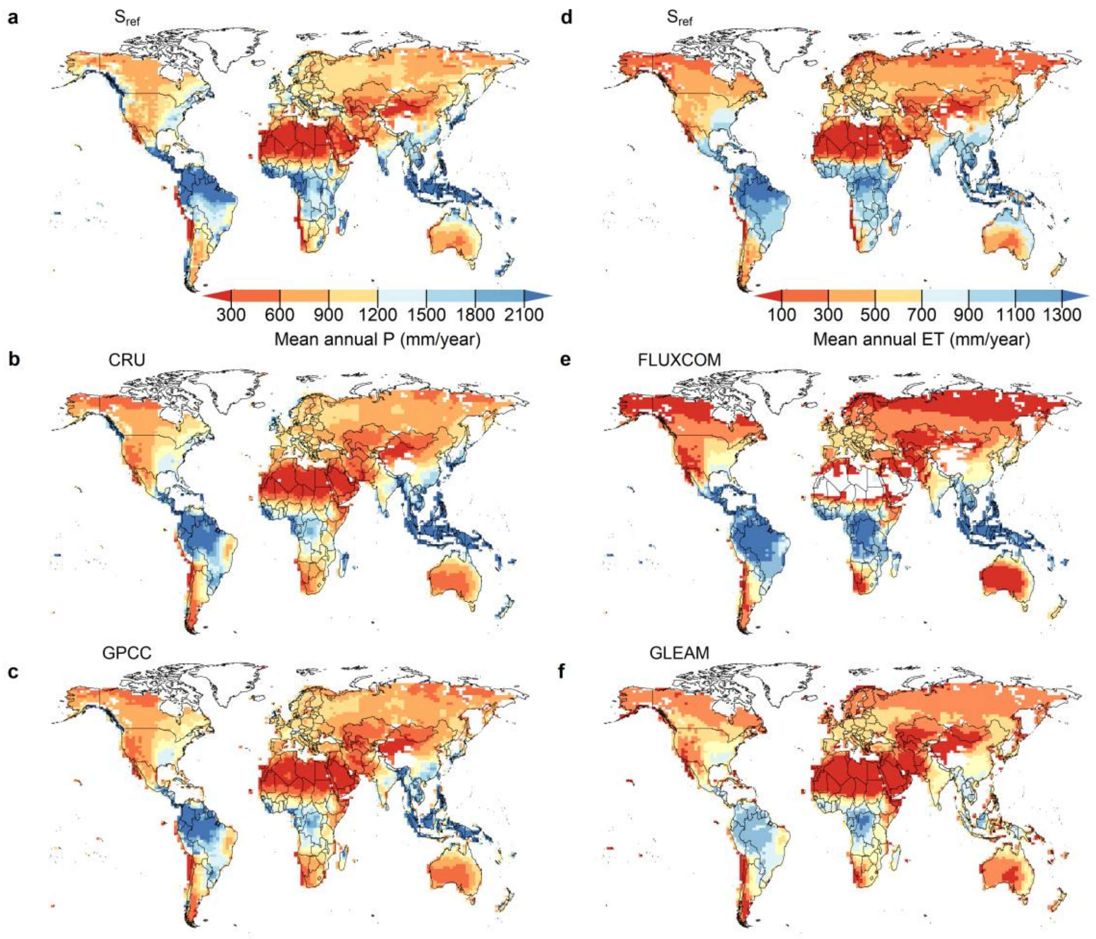

Figure S1 Spatial patterns of P (left) and ET (right) from the model outputs and the observation-based datasets. The model output from the reference simulation (S_{ref}) with present land cover is shown here for comparison. The details of the observation-based datasets are described in **Text S1.2**.

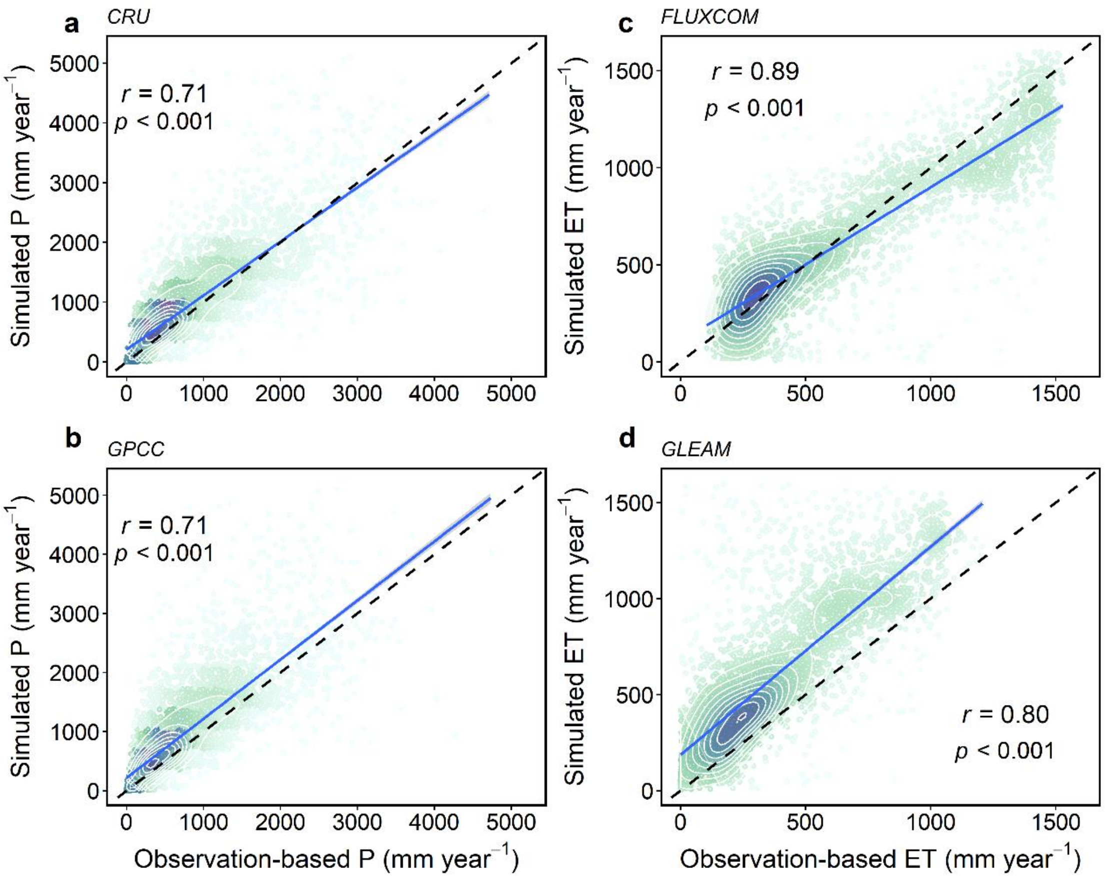

Figure S2 Scatter plots of simulated and observation-based P (left) and ET (right). Grid cells in various humidity provinces are shown as different colors. For the scatter plot, colored points and polygons show the kernel density of the studied points, with deeper colors indicating higher density distribution. The dashed line is the 1:1 ratio line. The blue line shows the linear regression between the simulated and the observation-based values and the correlation coefficients are given in each plot.

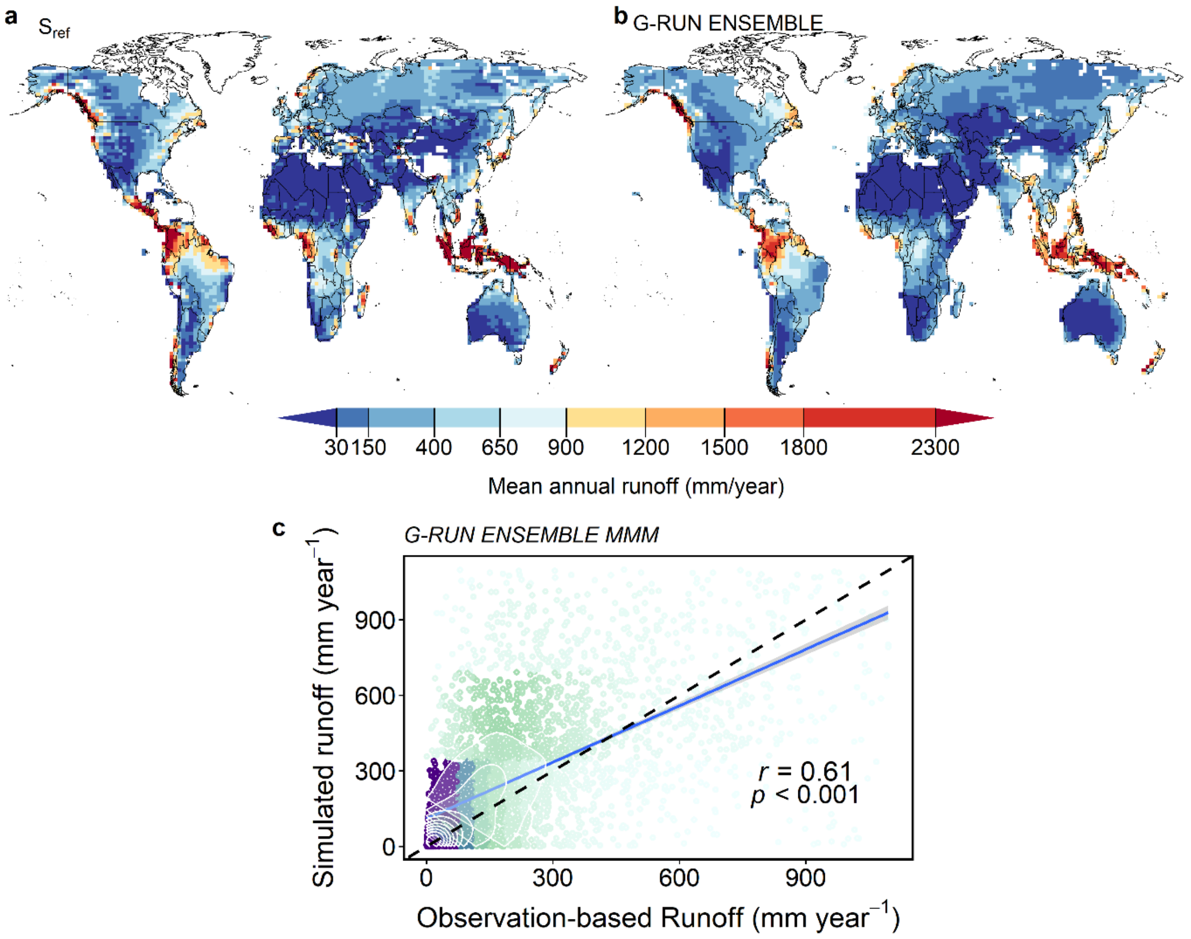

Figure S3 Model simulated and observation-based (G-RUN) runoff. Spatial patterns of the mean annual runoff derived from the model outputs (**a**) and the observation-based datasets (**b**). The scatter plot shows the paired comparison between simulated and observation-based runoff (**c**). Model outputs from the reference simulation (S_{ref}) with a present land cover map are used here. For the scatter plot, colored points and polygons show the kernel density of the studied points, with deeper colors indicating higher density distribution. The dashed line is the 1:1 ratio line. The blue line shows the linear regression between the simulated and the observation-based values and the correlation coefficients are given in each plot.

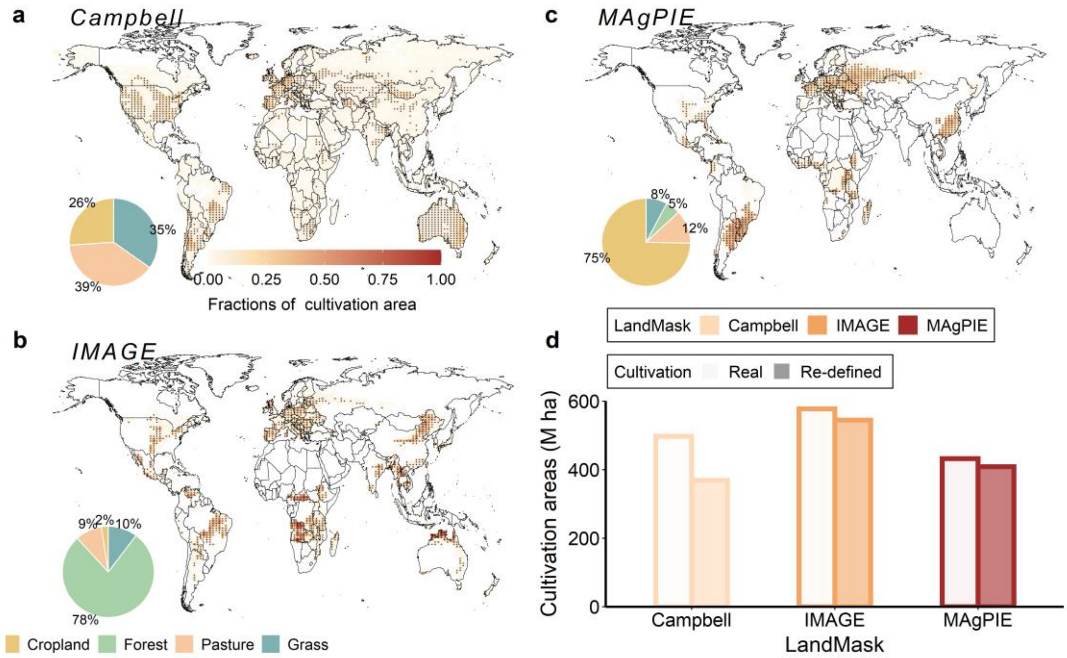

Figure S4 Bioenergy crop cultivation maps. Fractions of BECCS areas in each grid cell in the Campbell (**a**), IMAGE (**b**), MAgPIE (**c**) maps. Asterisks (*****) indicate grid cells with a fraction of BECCS areas of more than 0.05. The real used (empty columns) and redefined (filled columns) cultivation areas aggregated for each map (**d**). More details of the cultivation maps can be found in **Text S2**.

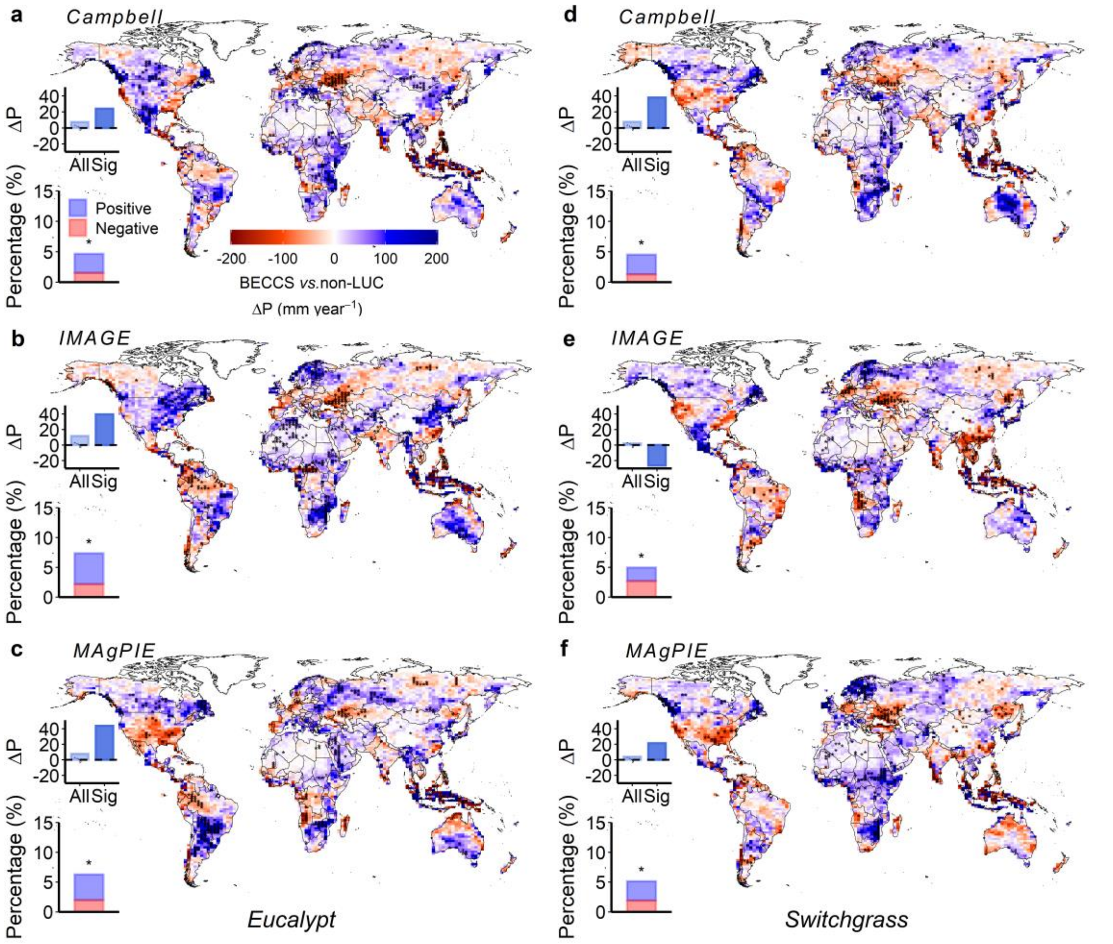

Figure S5 Spatial patterns of the changes in annual precipitation induced by eucalypt and switchgrass cultivation based on three cultivation maps. The changes in annual precipitation (ΔP) are calculated as the differences of the ten-year (i.e., the last ten years of the 50-year simulation period) annual precipitation between the BECCS scenarios and the reference (no land-use change) simulation. Spatial patterns of ΔP for eucalypt (**a, b, c**) and switchgrass (**d, e, f**) cultivation based on the cultivation maps of Campbell (the top row), IMAGE (the middle row) and MAgPIE (the bottom row). Stippling indicates that changes are statistically significant (according to the Wilcoxon signed-rank test at the 95% confidence level, $p < 0.05$). For each panel, the upper insets show the mean ΔP aggregated from all global land or the regions with significant changes. The lower insets show percentages of the global land area with significant changes detected, with positive changes shaded blue and negative changes shaded red.

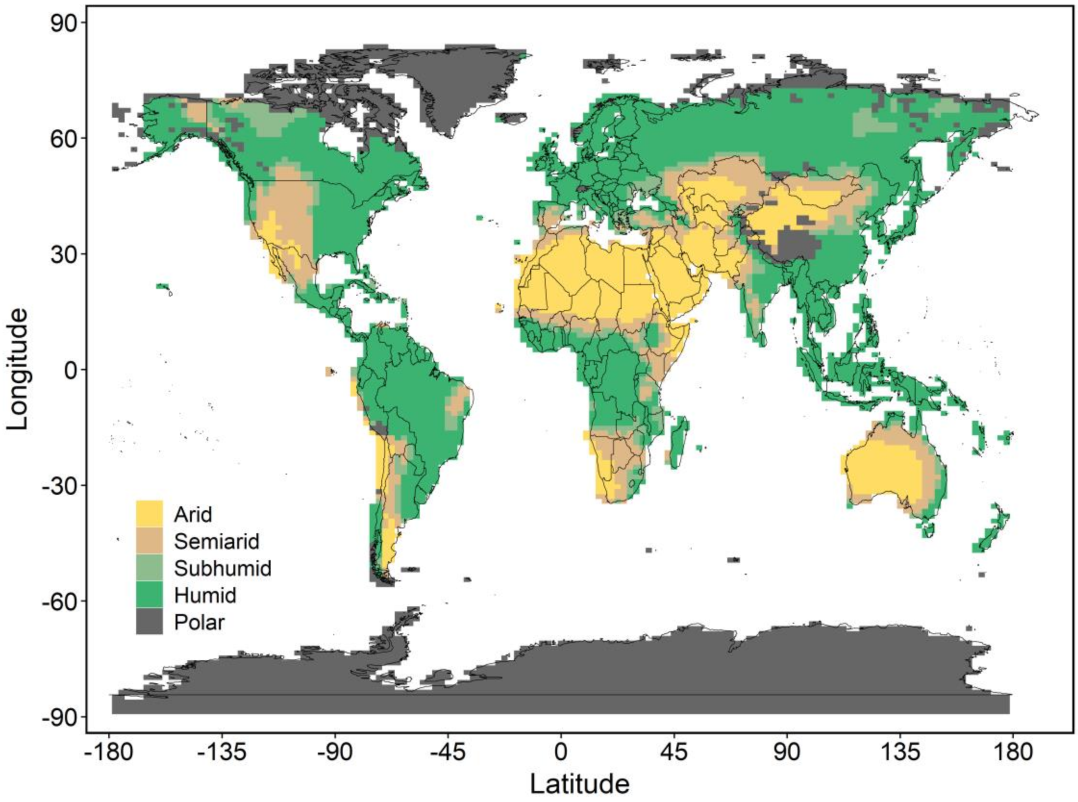

Figure S6 Humidity provinces based on the FAO aridity index, the Köppen-Geiger climate classification and the climatology annual precipitation. The division methods of the arid (yellow), semiarid (brown), subhumid (light green), humid (green) and polar (grey) zones are described in Methods.

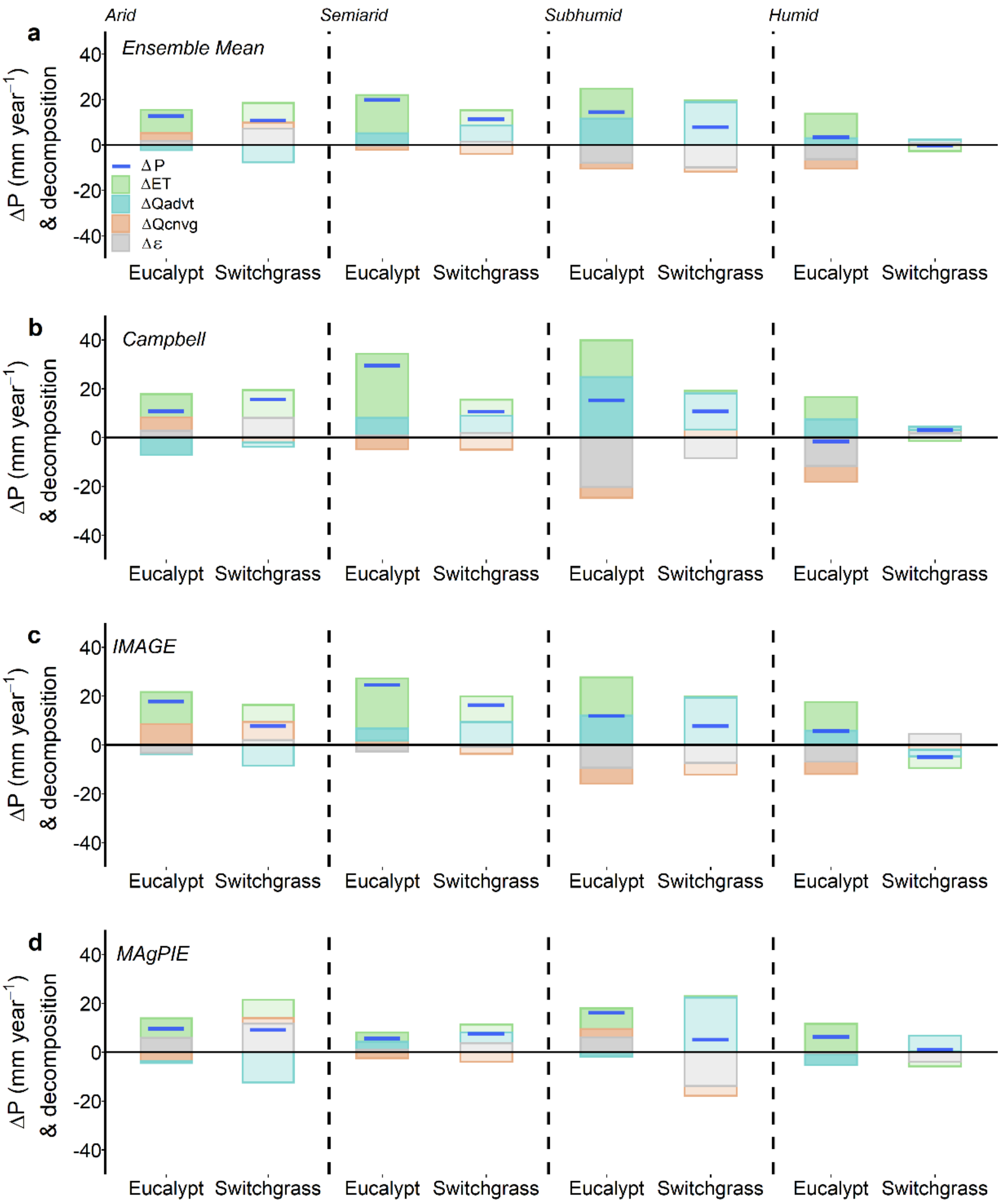

Figure S7 Precipitation changes and the diagnostic decomposition in each humidity province from simulations using different cultivation maps (b-d) and the ensemble mean (a). Stacked bars show the diagnostic decomposition of changed precipitation (ΔP, deep blue lines) from evapotranspiration (ΔET, light green columns), moisture advection (ΔQ_{advt}, light blue columns), and moisture convergence (ΔQ_{cnvg}, brick red columns) for eucalypt and switchgrass cultivation in the arid, semiarid, subhumid and humid zones. The residual term (Δε) is also plotted. Transparent columns show the switchgrass cultivation scenarios.

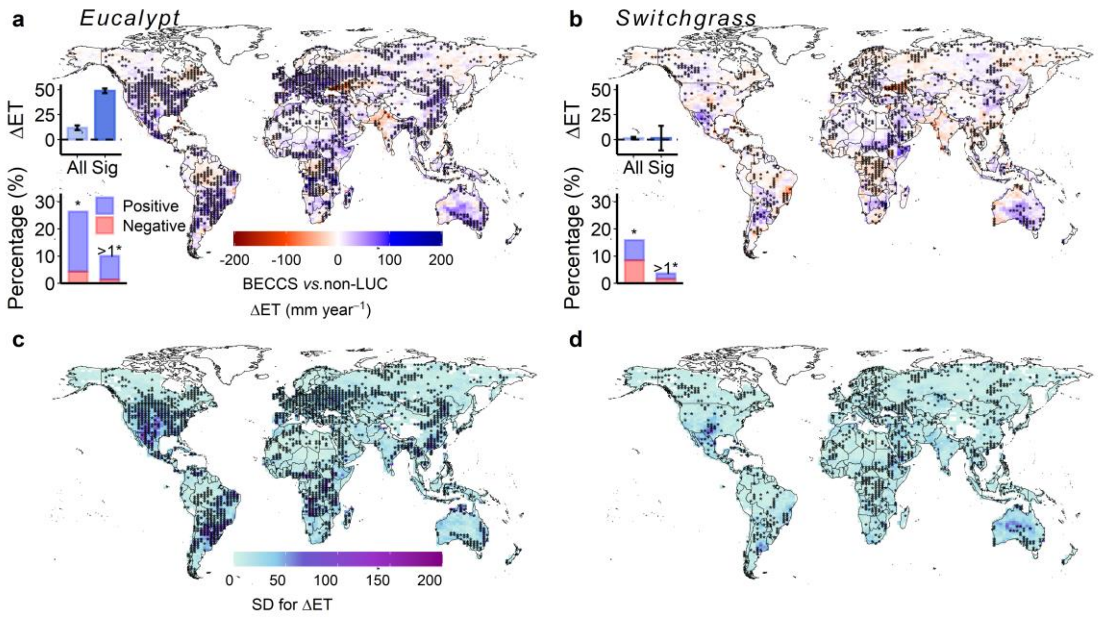

Figure S8 Spatial patterns of the changes in annual evapotranspiration induced by eucalypt and switchgrass cultivation. Changes in annual evapotranspiration (ΔET) are calculated as the differences of the ten-year (i.e., the last ten years of the 50-year simulation period) annual precipitation between BECCS scenarios and the reference (no land-use change) simulation. **a, b**, Spatial patterns of ΔET for eucalypt (**a**) and switchgrass (**b**) cultivations averaged over the tree cultivation maps (Fig. S4). **c, d**, Standard derivation (SD) of ΔET over the three cultivation scenarios for eucalypt (**c**) and switchgrass (**d**) cultivation. Stippling indicates that changes are statistically significant (according to the Wilcoxon signed-rank test at the 95% confidence level, $p < 0.05$) in at least one cultivation map. In panels **a** and **b**, the upper insets show mean and standard deviations of ΔP over the entire global land area (left) and over regions with statistically significant precipitation changes (right). The lower insets show percentages of the global land area with significant changes detected in one (left) or more than one (right) of the three cultivation maps, with positive changes shaded blue and negative changes shaded red.

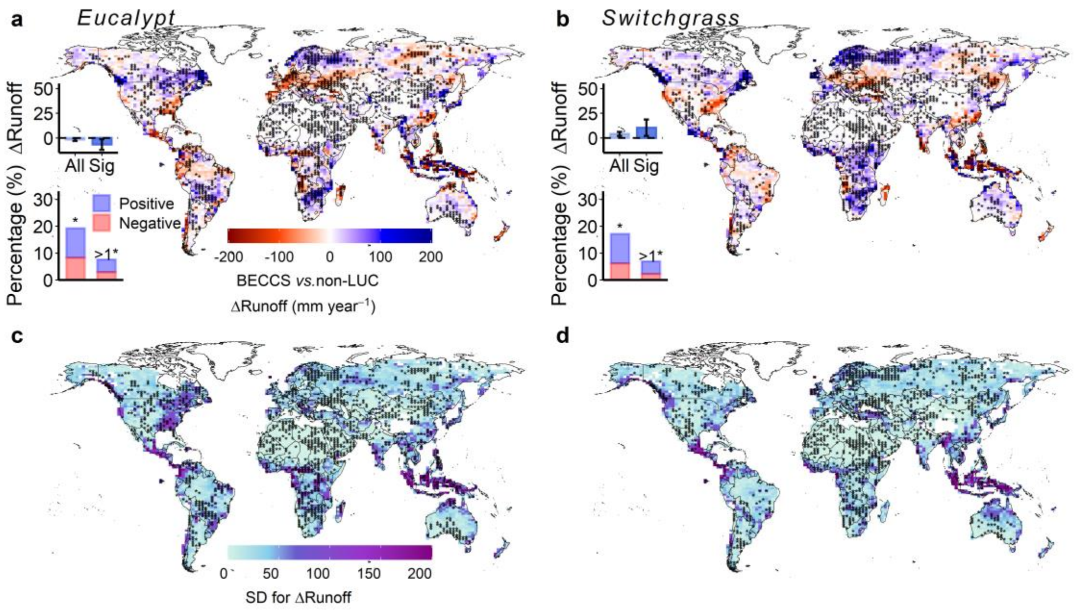

Figure S9 Spatial patterns of the changes in annual runoff induced by eucalypt and switchgrass cultivation. Changes in annual runoff (ΔRunoff) are calculated as the differences of the ten-year (i.e., the last ten years of the 50-year simulation period) annual precipitation between BECCS scenarios and the reference (no land-use change) simulation. **a, b**, Spatial patterns of ΔRunoff for eucalypt (**a**) and switchgrass (**b**) cultivation over the three cultivation maps (Fig. S4). **c, d**, Standard derivation (SD) of ΔRunoff over the three cultivation scenarios for eucalypt (**c**) and switchgrass (**d**) cultivation. Stippling indicates that changes are statistically significant (according to the Wilcoxon signed-rank test at the 95% confidence level, p <0.05) in at least one cultivation map. In panels **a** and **b**, the upper insets show mean and standard deviations of ΔP over the entire global land area (left) and over regions with statistically significant precipitation changes (right). The lower insets show percentages of the global land area with significant changes detected in one (left) or more than one (right) of the three cultivation maps, with positive changes shaded blue and negative changes shaded red.

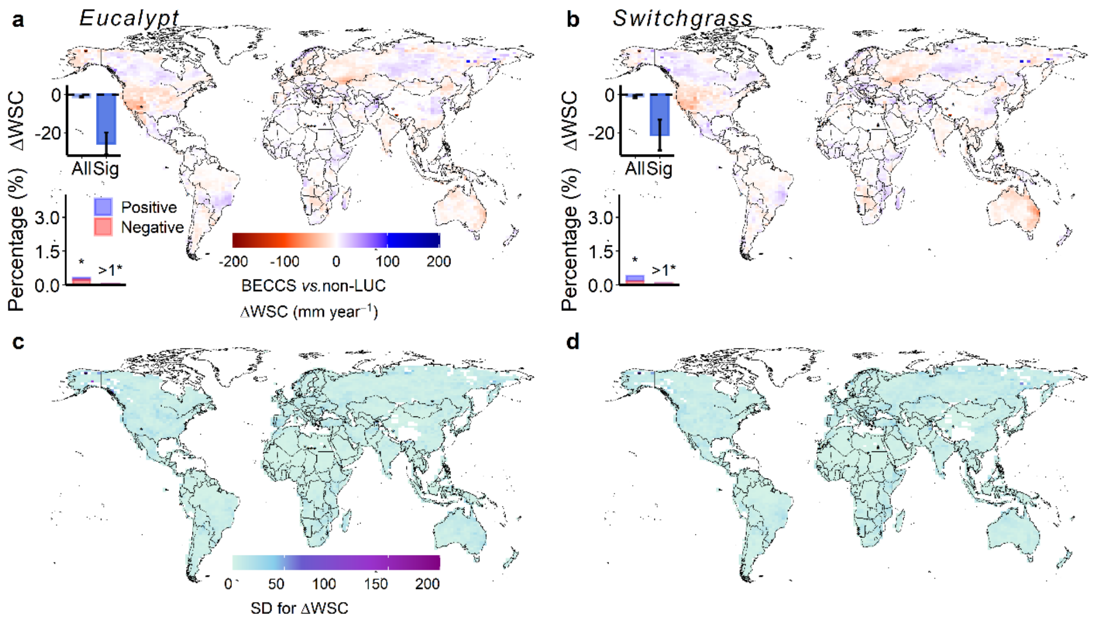

Figure S10 Spatial patterns of the changes in soil water storage changes induced by eucalypt and switchgrass cultivation. Changes in annual soil water storage changes (ΔWSC) are calculated as the differences of ten-year (i.e., the last ten years of the 50-year simulation period) annual WSC between BECCS scenarios and the reference (no land-use change) simulation. **a, b**, Spatial patterns of ΔWSC for eucalypt (**a**) and switchgrass (**b**) cultivation averaged over the three cultivation maps (Fig. S4). **c, d**, Standard derivation (SD) of ΔWSC over the three cultivation scenarios for eucalypt (**c**) and switchgrass (**d**) cultivation. Stippling indicates that changes are statistically significant (according to the Wilcoxon signed-rank test at the 95% confidence level, p <0.05) in at least one cultivation map. In panels **a** and **b**, the upper insets show mean and standard deviations of ΔP over the entire global land area (left) and over regions with statistically significant precipitation changes (right). The lower insets show percentages of the global land area with significant changes detected in one (left) or more than one (right) of the three cultivation maps, with positive changes shaded blue and negative changes shaded red.

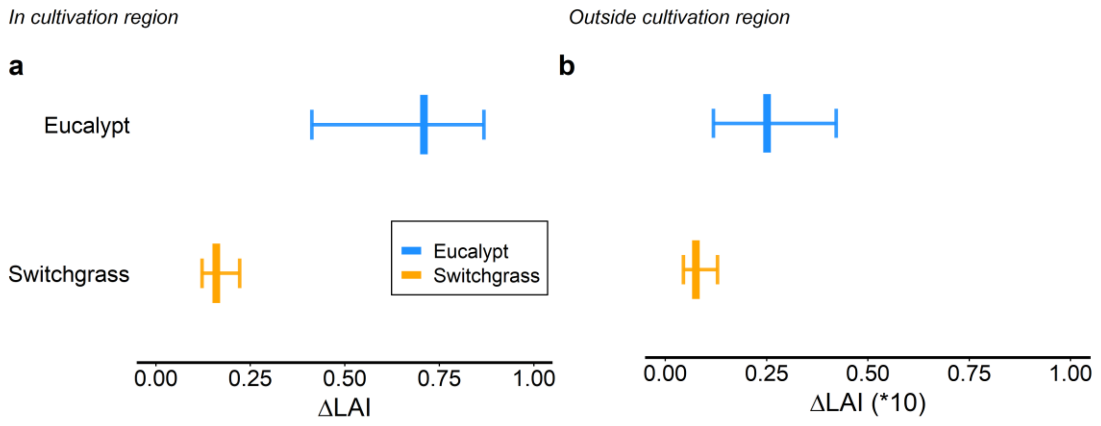

Figure S11 Changes in leaf area index (LAI) inside and outside the cultivation regions. Aggregated changes in mean annual LAI (ΔLAI) for eucalypt and switchgrass inside (**a**) and outside (**b**) the cultivation regions. The thick vertical lines and the error bars show the average and the range of ΔLAI across the three cultivation maps.

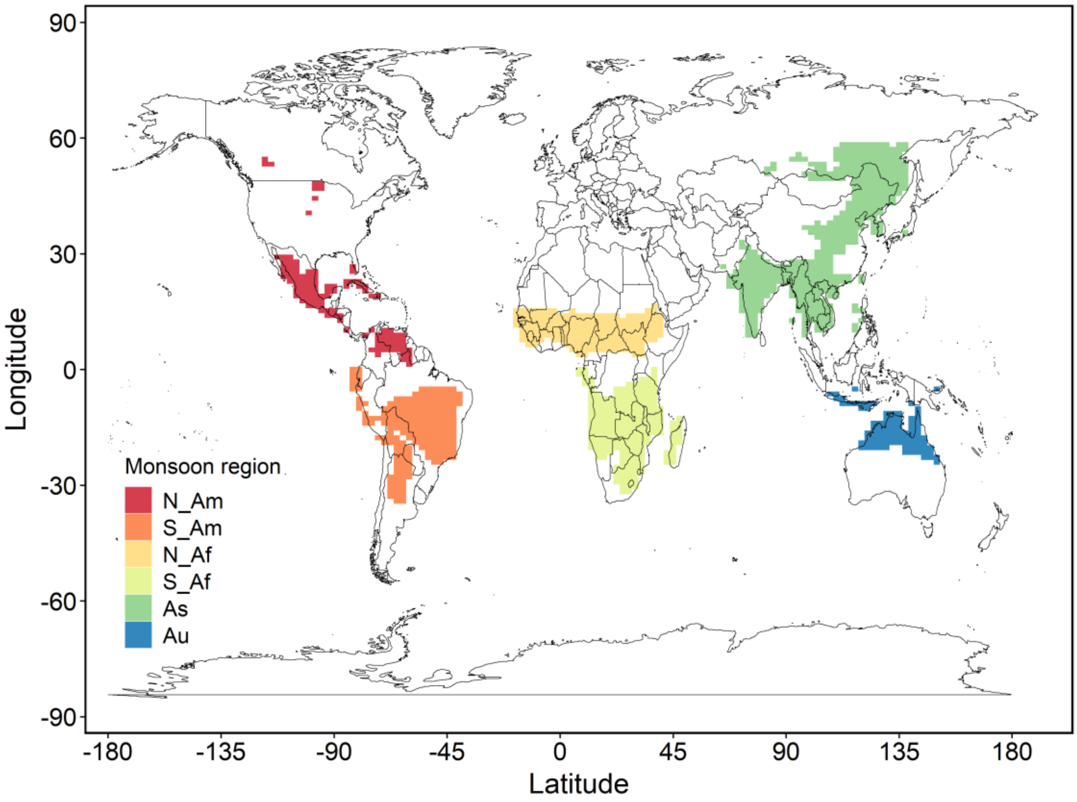

Figure S12 Map of the monsoon area. The six monsoon regions are the North American (N_Am), South American (S_Am), North African (N_Af), South African (S_Af), Asian (As) and Australian (Au) monsoon regions.

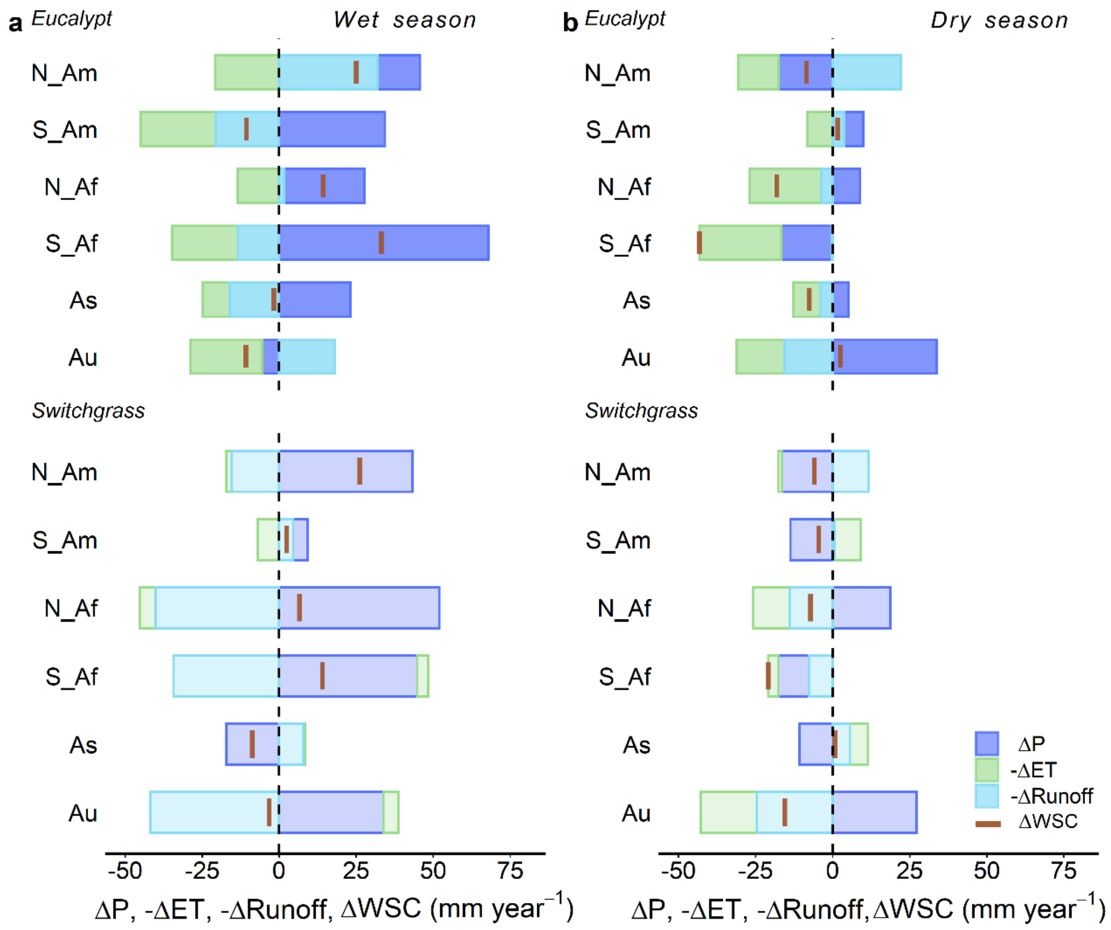

Figure S13 Changes in terrestrial water balance components for the six monsoon regions induced by large-scale bioenergy crop cultivation. Changes in precipitation (ΔP), evapotranspiration (ΔET), runoff (ΔRunoff) and water storage (ΔWSC) for the eucalypt and switchgrass cultivation scenarios in the six monsoon regions during the wet (**a**) and dry (**b**) seasons. Note that ΔET and ΔRunoff are shown with reversed signs (-ΔET and -ΔRunoff) to be consistent with the ΔWSC deduced from the water balance (Eq. 5).

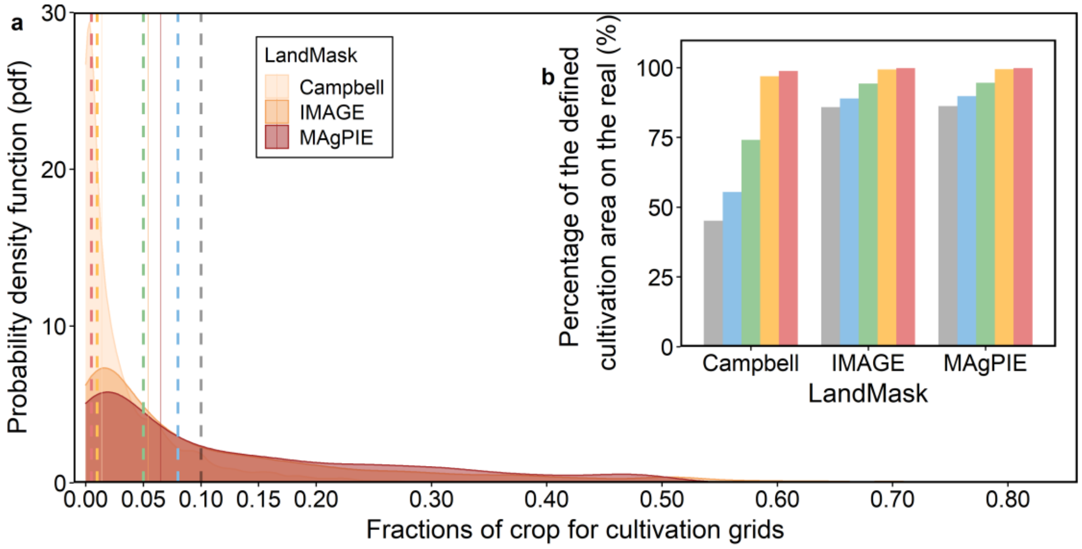

Figure S14 Evaluation of the fraction of crop land (f_{Bcrop}) on the bioenergy crop cultivation areas. a, Probability density function (pdf) of f_{Bcrop} for land-use maps, Campbell, IMAGE and MAgPIE. Solid lines show the median value of each land-use map in corresponding colors. The dashed lines show the evaluation of f_{crop} of 0.1 (grey), 0.08 (blue), 0.05 (green), 0.01 (yellow), 0.005 (rose red).

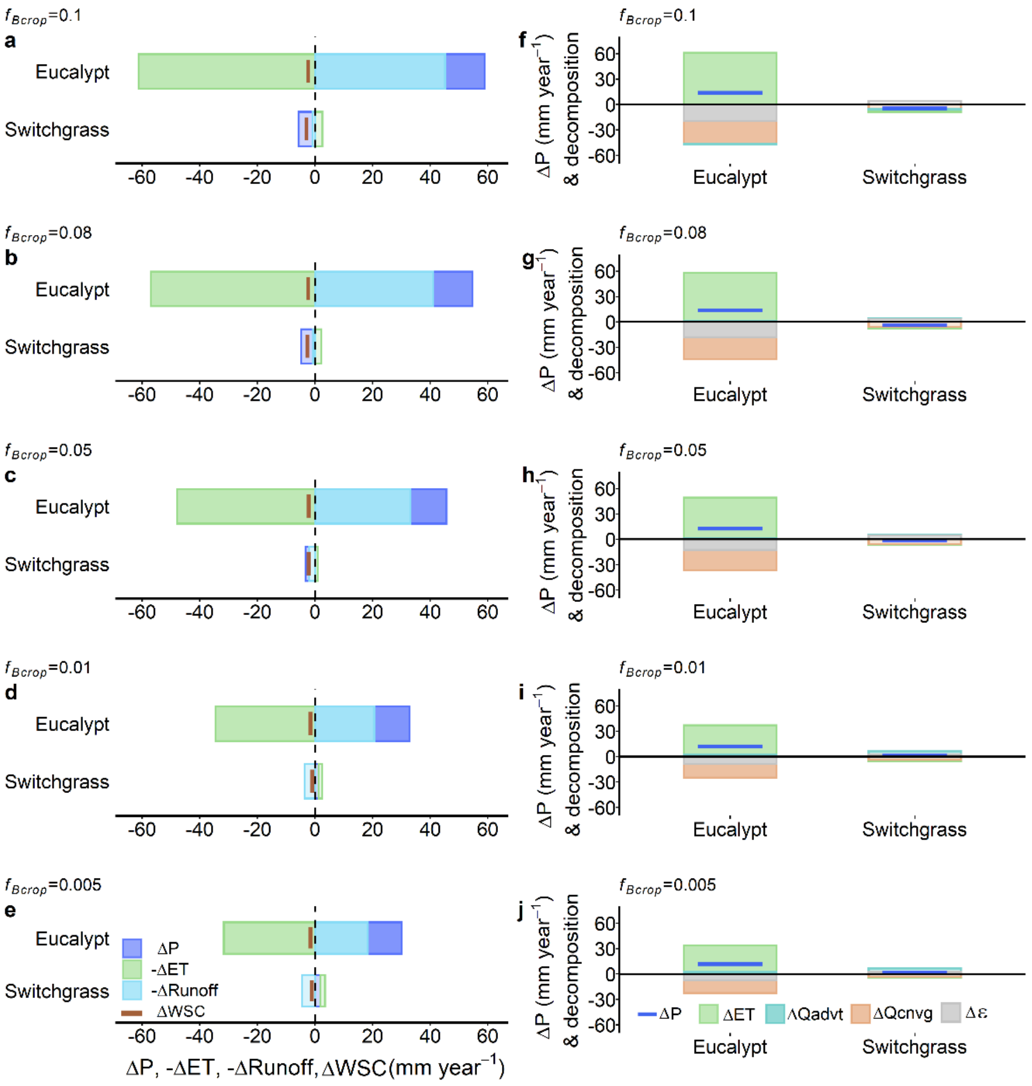

Figure S15 Changes in terrestrial water balance components and diagnostic precipitation changes in the cultivation regions defined by different f_{Bcrop} (fraction thresholds). (a-e) Aggregated changes in precipitation (ΔP), evapotranspiration (ΔET), runoff (ΔRunoff) and soil water storage (ΔWSC) for eucalypt and switchgrass cultivation over three cultivation maps in the cultivation regions defined by different values of f_{Bcrop}. Note that, ΔET and ΔRunoff are shown with reversed signs (-ΔET and -ΔRunoff) to be consistent with the ΔWSC deduced from the water balance (Eq. 5). **(f-j)** Diagnosis for precipitation changes (ΔP, deep blue lines) from changes in evapotranspiration (ΔET, light green columns), moisture advection (ΔQ_{advt}, light blue columns) and moisture convergence (ΔQ_{cnvg}, brick red columns) for eucalypt and switchgrass cultivation over three cultivation maps in the cultivation regions defined by different thresholds of f_{Bcrop} (The residual terms, Δε, are also plotted with olive green columns). Transparent columns show the switchgrass cultivation scenario.

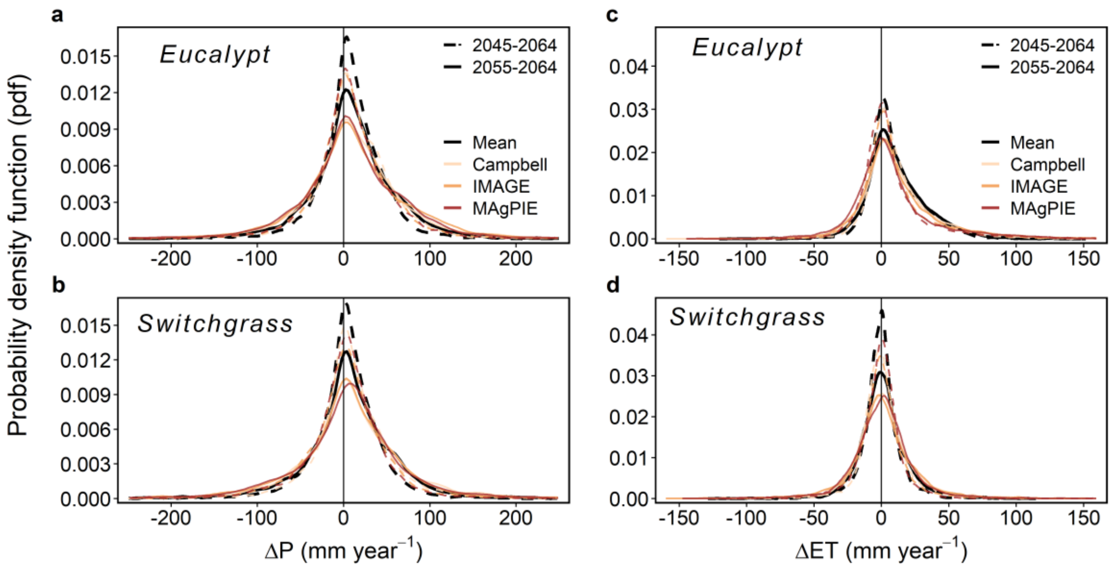

Figure S16 Probability density function (pdf) of the changes in P and ET from different simulation periods. Colored lines show the results from each cultivation map, and black lines indicate the ensemble mean.

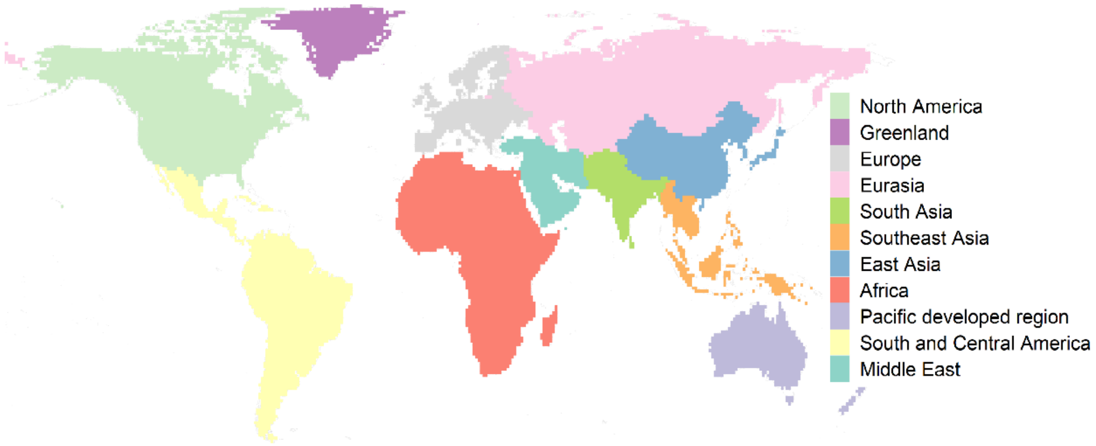

Figure S17. The region divisions used in the analysis of the most suitable bioenergy crop.

Table S1 Classification of the humidity provinces

Humidity province	Aridity Index (AI)	K-G Classifications	Data sources
Arid	AI <0.2	-	FAO, Aridity index (FAO, 2015)[47]
Semiarid	0.2 ≤AI< 0.5	-	FAO, Aridity index (FAO, 2015)[47]
Subhumid	0.5 ≤AI < 0.65	-	FAO, Aridity index (FAO, 2015)[47]
Humid	AI ≥0.65	-	FAO, Aridity index (FAO, 2015)[47]
Polar	-	ET, EF	Köppen-Geiger classifications for present-day maps (Beck et al., 2018)[48]

Table S2. "Local" contributions to changes in precipitation (ΔP_{local}) by ET and moisture convergence (Q_{cnvg}) in each region (region division in Fig. S17) in the eucalypt and switchgrass scenarios.

	ΔP_{local} (mm year^{-1})	
	Eucalypt	Switchgrass
North America	14.7±15.6	-2.9±6.6
Europe	13.3±9.1	0.4±3.9
Eurasia	4.4±4.3	1.4±2.2
South Asia	6.9±21.1	-17.7±6.7
Southeast Asia	-21.3±30.6	-24.5±17.7
East Asia	19.8±21.6	-18.5±3.5
Africa	10.3±11.7	8.7±0.4
Pacific developed region	25.9±18.7	10.8±28.5
South and Central America	15.5±13.0	10.1±1.8
Middle East	32.4±20.2	38.0±12.5

Supplementary References

1 Hourdin, F. et al. LMDZ6A: The Atmospheric Component of the IPSL Climate Model With Improved and Better Tuned Physics. *J. Adv. Model. Earth. Sy.* **12**, e2019MS001892 (2020).

2 Li, W. et al. ORCHIDEE-MICT-BIOENERGY: an attempt to represent the production of lignocellulosic crops for bioenergy in a global vegetation model. *Geosci. Model. Dev.* **11**, 2249-2272 (2018).

3 Hourdin, F. et al. LMDZ5B: the atmospheric component of the IPSL climate model with revisited parameterizations for clouds and convection. *Clim. Dynam.* **40**, 2193-2222 (2013).

4 Boucher, O. et al. Presentation and evaluation of the IPSL‐CM6A‐LR climate model. *J. Adv. Model. Earth. Sy.* **12**, e2019MS002010 (2020).

5 Zeng, Z. et al. Climate mitigation from vegetation biophysical feedbacks during the past three decades. *Nat. Clim. Change* **7**, 432-436 (2017).

6 Peng, S.-S. et al. Afforestation in China cools local land surface temperature. *P. Natl. Acad. Sci. USA* **111**, 2915-2919 (2014).

7 Guimberteau, M. et al. ORCHIDEE-MICT (v8.4.1), a land surface model for the high latitudes: model description and validation. *Geosci. Model Dev.* **11**, 121-163 (2018).

8 Krinner, G. et al. A dynamic global vegetation model for studies of the coupled atmosphere-biosphere system. *Global Biogeochem. Cy.* **19**, 1–33 (2005).

9 Yue, C. et al. Representing anthropogenic gross land use change, wood harvest, and forest age dynamics in a global vegetation model ORCHIDEE-MICT v8.4.2. *Geosci. Model Dev.* **11**, 409-428 (2018).

10 Li, Y. et al. Divergent hydrological response to large-scale afforestation and vegetation greening in China. *Sci. Adv.* **4**, eaar4182 (2018).

11 Mueller, B. & Seneviratne, S. I. Systematic land climate and evapotranspiration biases in CMIP5 simulations. *Geophys. Res. Lett.* **41**, 128-134 (2014).

12 Wang, J. et al. Global cooling induced by biophysical effects of bioenergy crop cultivation. *Nature Commun.* **12**, 1-9 (2021).

13 Harris, I., Osborn, T. J., Jones, P. & Lister, D. Version 4 of the CRU TS monthly high-resolution gridded multivariate climate dataset. *Sci. Data* **7**, 109 (2020).

14 Markus, Z. et al. GPCC Full Data Daily Version 2020 at 1.0°: Daily Land-Surface Precipitation from Rain-Gauges built on GTS-based and Historic Data. DOI: 10.5676/DWD_GPCC/FD_D_V2020_100 (2020).

15 Jung, M. et al. The FLUXCOM ensemble of global land-atmosphere energy fluxes. *Sci. Data* **6**, 74 (2019).

16 Martens, B. et al. GLEAM v3: satellite-based land evaporation and root-zone soil moisture. *Geosci. Model Dev.* **10**, 1903-1925 (2017).

17 Harris, I., Jones, P. D., Osborn, T. J. & Lister, D. H. Updated high-resolution grids of monthly climatic observations – the CRU TS3.10 Dataset. *Int. J. Climatol.* **34**, 623-642 (2014).

18 Becker, A. et al. A description of the global land-surface precipitation data products of the Global Precipitation Climatology Centre with sample applications including centennial (trend) analysis from 1901–present. *Earth Syst. Sci. Data* **5**, 71-99 (2013).

19 Tramontana, G. et al. Predicting carbon dioxide and energy fluxes across global FLUXNET sites with regression algorithms. *Biogeosciences* **13**, 4291-4313 (2016).

20 Miralles, D. G. et al. Global land-surface evaporation estimated from satellite-based observations. *Hydrol. Earth Syst. Sci.* **15**, 453-469 (2011).

21 Ghiggi, G., Humphrey, V., Seneviratne, S. I. & Gudmundsson, L. G - RUN ENSEMBLE: A Multi - Forcing Observation - Based Global Runoff Reanalysis. *Water Resour. Res.* **57**, e2020WR028787 (2021).

22 Gudmundsson, L., Do, H. X., Leonard, M. & Westra, S. The Global Streamflow Indices and Metadata Archive (GSIM)–Part 2: Quality control, time-series indices and homogeneity assessment. *Earth Syst. Sci. Data* **10**, 787-804 (2018).

23 Do, H. X., Gudmundsson, L., Leonard, M. & Westra, S. The Global Streamflow Indices and Metadata Archive (GSIM)–Part 1: The production of a daily streamflow archive and metadata. *Earth Syst. Sci. Data* **10**, 765-785 (2018).

24 Warszawski, L. et al. The inter-sectoral impact model intercomparison project (ISI–MIP): project framework. *P. Natl. Acad. Sci. USA* **111**, 3228-3232 (2014).

25 Schmer, M. R., Vogel, K. P., Mitchell, R. B. & Perrin, R. K. Net energy of cellulosic ethanol from switchgrass. *P. Natl. Acad. Sci. USA* **105**, 464-469 (2008).

26 Campbell, J. E., Lobell, D. B., Genova, R. C. & Field, C. B. The global potential of bioenergy on abandoned agriculture lands. *Environ. Sci. Technol.* **42**, 5791-5794 (2008).

27 Goldewijk, K. K. Estimating global land use change over the past 300 years: the HYDE database. *Global Biogeochem. Cy.* **15**, 417-433 (2001).

28 Friedl, M. A. et al. Global land cover mapping from MODIS: algorithms and early results. *Remote Sens. Environ.* **83**, 287-302 (2002).

29 Stehfest, E., van Vuuren, D., Bouwman, L. & Kram, T. Integrated assessment of global environmental change with IMAGE 3.0: Model description and policy applications. PBL Netherlands Environmental Assessment Agency (2014).

30 Dietrich, J. P. et al. MAgPIE 4–a modular open-source framework for modeling global land systems. *Geosci. Model Dev.* **12**, 1299-1317 (2019).

31 Popp, A. et al. Land-use futures in the shared socio-economic pathways. *Global Environ. Chang.* **42**, 331-345 (2017).

32 Riahi, K. et al. The Shared Socioeconomic Pathways and their energy, land use, and greenhouse gas emissions implications: An overview. *Global Environ. Chang.* **42**, 153-168 (2017).

33 Klein Goldewijk, K., Beusen, A., Van Drecht, G. & De Vos, M. The HYDE 3.1 spatially explicit database of human-induced global land-use change over the past 12,000 years. *Global Ecol. Biogeogr.* **20**, 73-86 (2011).

34 Krause, A. et al. Global consequences of afforestation and bioenergy cultivation on ecosystem service indicators. *Biogeosciences* **14**, 4829-4850 (2017).

35 Li, W. et al. Mapping the yields of lignocellulosic bioenergy crops from observations at the global scale. *Earth Syst. Sci. Data* **12**, 789-804 (2020).

36 Starr, V. P. & Peixoto, J. P. On the Global Balance of Water Vapor and the Hydrology of Deserts. *Tellus* **10**, 188-194 (1958).

37 Peixoto, J. P. & Oort, A. H. *Physics of climate* (American Institute of Physics, 1992).

38 Rasmusson, E. M. Atmospheric water vapor transport and the water balance of North America: II. Large-scale water balance investigations. *Mon. Weather Rev.* **96**, 720-734 (1968).

39 Mariotti, A., Struglia, M. V., Zeng, N. & Lau, K.-M. The Hydrological Cycle in the Mediterranean Region and Implications for the Water Budget of the Mediterranean Sea. *J. Climate* **15**, 1674-1690 (2002).

40 Wang, Y., Zhang, G. J. & Jiang, Y. Linking stochasticity of convection to large-scale vertical velocity to improve Indian summer monsoon simulation in the NCAR CAM5. *J. Climate* **31**, 6985-7002 (2018).

41 Wang, T., Wong, S. & Fetzer, E. J. Cloud regime evolution in the Indian monsoon intraseasonal oscillation: Connection to large-scale dynamical conditions and the atmospheric water budget. *Geophys. Res. Lett.* **42**, 9465-9472 (2015).

42 Chang, C.P. The global monsoon system: research and forecast. *World Scientific* **5**, (2011).

43 Wang, B. & Ding, Q. Global monsoon: Dominant mode of annual variation in the tropics. *Dynam. Atmos. Oceans* **44**, 165-183 (2008).

44 Hsu, P. C. et al. Increase of global monsoon area and precipitation under global warming: A robust signal? *Geophys. Res. Lett*. **39**, GL051037 (2012).

45 Liu, J. et al. Centennial Variations of the Global Monsoon Precipitation in the Last Millennium: Results from ECHO-G Model. *J. Climate* **22**, 2356-2371 (2009).

46 Wang, B. & Ding, Q. Changes in global monsoon precipitation over the past 56 years. *Geophys. Res. Lett*. **33**, GL025347 (2006).

47 Food and Agriculture Organization of the United Nations, FAO GEONETWORK. Global map of aridity-10 arc minutes (GeoLayer), FAO (2015).

48 Beck, H. E. et al. Present and future Köppen-Geiger climate classification maps at 1-km resolution. *Sci. Data* **5**, 1-12 (2018).
